# Cooperative Binding of the Cationic Porphyrin Tris-T4 Enhances Catalytic Activity of 20S Proteasome Unveiling a Complex Distribution of Functional States

**DOI:** 10.3390/ijms21197190

**Published:** 2020-09-29

**Authors:** Anna Maria Santoro, Alessandro D’Urso, Alessandra Cunsolo, Danilo Milardi, Roberto Purrello, Diego Sbardella, Grazia R. Tundo, Donatella Diana, Roberto Fattorusso, Antonio Di Dato, Antonella Paladino, Marco Persico, Massimo Coletta, Caterina Fattorusso

**Affiliations:** 1 Istituto di Cristallografia—CNR Sede Secondaria di Catania, Via P. Gaifami 9/18, 95126 Catania, Italy; amsantoro@unict.it (A.M.S.); dmilardi@dipchi.unict.it (D.M.); 2 Dipartimento di Scienze Chimiche, Università Degli Studi di Catania, Viale A. Doria 6, 95125 Catania, Italy; adurso@unict.it (A.D.); acunsolo@unict.it (A.C.); rpurrello@dipchi.unict.it (R.P.); 3 Department of Molecular Medicine, The University of Texas Health Science Center San Antonio, 7703 Floyd Curl Drive, San Antonio, TX 78245, USA; 4 IRCCS-Fondazione Bietti, 00198 Rome, Italy; diego.sbardella@fondazionebietti.it (D.S.); graziaraffaella.tundo@fondazionebietti.it (G.R.T.); 5 Istituto di Biostrutture e Bioimmagini, CNR, Via Mezzocannone 16, 80134 Napoli, Italy; donatelladiana@gmail.com; 6 Department of Environmental, Biological and Pharmaceutical Sciences and Technologies, University of Campania “Luigi Vanvitelli” Via Vivaldi 43, 81100 Caserta, Italy; roberto.fattorusso@unicampania.it; 7 Dipartimento di Farmacia, Università di Napoli “Federico II”, Via D. Montesano 49, 80131 Napoli, Italy; antonio.didato@unina.it (A.D.D.); m.persico@unina.it (M.P.); 8 Istituto di Chimica del Riconoscimento Molecolare, CNR, Via M. Bianco 9, 20131 Milano, Italy; paladino.anto@gmail.com; 9 Centro Interuniversitario di Ricerca sulla Malaria/Italian Malaria Network, 80131 Napoli, Italy; 10 Dipartimento di Scienze Cliniche e Medicina Traslazionale, Università di Roma Tor Vergata, Via Montpellier 1, 00133 Roma, Italy

**Keywords:** 20S proteasome, allosteric modulator, kinetic analysis, conformational/functional equilibria, NMR studies, molecular dynamics simulation, integrated interaction model

## Abstract

The present study provides new evidence that cationic porphyrins may be considered as tunable platforms to interfere with the structural “key code” present on the 20S proteasome α-rings and, by consequence, with its catalytic activity. Here, we describe the functional and conformational effects on the 20S proteasome induced by the cooperative binding of the tri-cationic 5-(phenyl)-10,15,20-(tri *N*-methyl-4-pyridyl) porphyrin (Tris-T4). Our integrated kinetic, NMR, and in silico analysis allowed us to disclose a complex effect on the 20S catalytic activity depending on substrate/porphyrin concentration. The analysis of the kinetic data shows that Tris-T4 shifts the relative populations of the multiple interconverting 20S proteasome conformations leading to an increase in substrate hydrolysis by an allosteric pathway. Based on our Tris-T4/h20S interaction model, Tris-T4 is able to affect gating dynamics and substrate hydrolysis by binding to an array of negatively charged and hydrophobic residues present on the protein surface involved in the 20S molecular activation by the regulatory proteins (RPs). Accordingly, despite the fact that Tris-T4 also binds to the α3ΔN mutant, allosteric modulation is not observed since the molecular mechanism connecting gate dynamics with substrate hydrolysis is impaired. We envisage that the dynamic view of the 20S conformational equilibria, activated through cooperative Tris-T4 binding, may work as a simplified model for a better understanding of the intricate network of 20S conformational/functional states that may be mobilized by exogenous ligands, paving the way for the development of a new generation of proteasome allosteric modulators.

## 1. Introduction

The ubiquitin-proteasome system (UPS) is the major cytosolic proteolytic machinery and its regulation has a profound impact on all cellular metabolic pathways [1]. The central element of the UPS is the 20S proteasome core particle (CP), a multi-subunit protease responsible for the degradation of protein substrates [2]. Although the proteasome has been extensively investigated as drug target [3,4] and CP catalytic inhibitors have been approved by FDA for the treatment of multiple myeloma, our knowledge of the molecular mechanisms underlying its function is still far from comprehensive. This impairs the development of a second-generation of proteasome inhibitors to overcome the drawbacks of the existing drugs (such as the induction of resistance), as well as the development of new proteasome-based therapeutic strategies for cancer and many other diseases, including those related to protein conformational disorders (PCD). 

**Figure 1:**
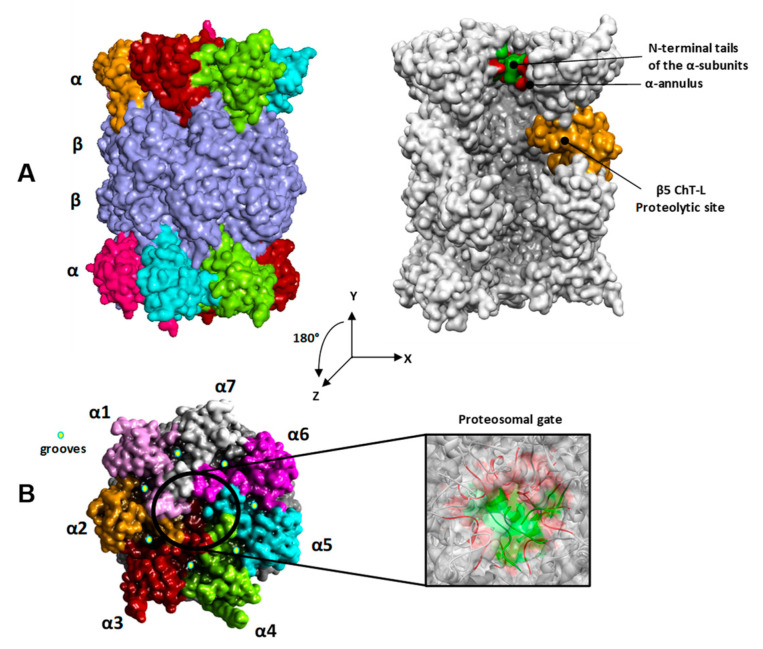
(**A**) Transversal and (**B**) top views of the h20S proteasome (closed form; PDB ID: 4R3O). Left: the α subunits are colored in pink (α1), orange (α2), brown (α3), light green (α4), cyan (α5), magenta (α6), and grey (α7); the β subunits are colored in violet. Right: N-terminal tails of the α subunits (i.e., α2–α4) are evidenced in green; the loops of the α subunits forming the α-annulus are evidenced in red; the catalytic β5 subunit is colored in orange. For clarity of presentation just a cross section of h20S proteasome is shown in A (right).

The first CP structure has been solved by X-ray crystallography more than two decades ago [5]. It is composed by 28 subunits arranged in four rings (α1-7, β1-7, β1-7, α1-7), with a symmetric, cylinder shaped, heterodimeric structure (Figure 1A) where three couples of chymotrypsin-like (ChT-L), trypsin-like (T-L), and caspase-like (C-L) proteolytic sites are located. The substrate access to the β-subunit catalytic sites is limited by the N-terminal tails of the α-subunits forming a gate which occludes the entrance to the narrow pore in the α-ring (α-annulus) (Figure 1A,B, right) [6]. 

The solvent-exposed surface of the α-rings is structurally characterized by the presence of shallow grooves between the α-subunits serving as anchor points for the regulatory particles (RPs) (Figure 1B, left) [7,8,9,10,11,12,13,14,15]. The human 20S CP (h20S) may interact with one or two 19S RPs, forming the 26S or the 30S complex, respectively [16,17]. Besides 19S, h20S may bind other RPs, such as, PA28 [13,18] and PA200 [19]. The structural information acquired by X-ray crystallography [13,14], NMR [20], Cryo-EM [7,8,9,10,11,12], and biochemical studies [21,22] pointed out that RPs induce specific 20S conformational changes, thus modulating the allosteric mechanism by which protein substrates are translocated and hydrolyzed into the catalytic sites.

Some substrates, such as mutated, oxidized, and intrinsically disordered proteins (IDP), are preferentially degraded through an RP-independent process, mediated by the naked catalytic 20S CP [23,24]. In the absence of RPs, the gate formed by the flexible N-terminal tails of the α-subunits play a major role in regulating the entrance of substrates to the central channel, suggesting a substrate-dependent transition between a “closed” and an “open” state in latent 20S. Apart from the substrate, a conserved motif (HbYX; hydrophobic, Hb; Tyrosine, Y; any amino acid, X) at the C-terminal tails of 19S and PA200 RPs was proved to be able to activate 20S catalytic activity [15,25,26,27]. Moreover, positively charged peptides or chemicals were also reported to act as 20S allosteric modulators, being able to either activate or inhibit its catalytic activity e.g., PA28 derived peptides [28], HIV-1 Tat derived peptides [28,29], PR39 derived peptides [30], the octa peptide Arg(8) [31] and citicoline, a neuro-protective drug used in ophthalmology [32]. Structure-activity relationships (SARs) studies pointed out the complex interplay existing between structural flexibility and charge in the regulation of the inhibitory potency of Tat-derived peptides [33]. Ionic interactions are also at the base of the allosteric modulation of the h20S catalytic activities by insulin degrading enzyme (IDE) [34,35].

**Figure 2:**
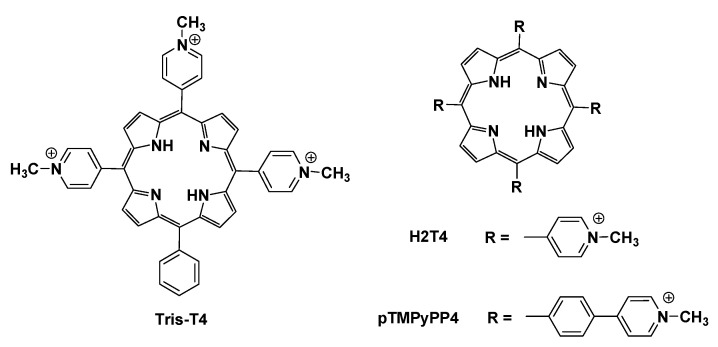
Chemical structure of porphyrins H2T4, Tris-T4 and pTMPyPP4.

Within this frame, we previously reported that the number and the orientation of the positively charged groups on cationic porphyrins are crucial for their effect on the enzymatic activity of 20S [36,37,38]. We identified a cluster of four negatively charged residues lining the h20S substrate gate as the putative binding site for the poly-aromatic and positively charged structure of the meso-tetrakis (4-*N*-methylpyridyl)-porphyrin (H2T4; Figure 2) [37], a h20S competitive inhibitor discovered by us [36]. Then, an in-depth structural ad bioinformatic analysis revealed the existence on h20S α-rings of a sort of ionic code for the interaction with the charged residues present on RPs [38]. On these bases, starting from the structure of the competitive inhibitor H2T4, just changing the distribution of the four positive charges around the rigid porphyrin scaffold, we obtained the allosteric inhibitor meso-tetrakis (4-*N*-methylphenyl-pyridyl)-porphyrin (pTMPyPP4; Figure 2) [38]. This result strongly supported the putative ability of cationic porphyrins to partially reproduce the orientation of the positively charged residues present on the RPs, matching the ionic code on the 20S surface.

Taken as a whole, these data suggested that cationic porphyrins may be considered as tunable platforms to interfere with the structural “key code” present on the surface of 20S proteasome responsible for the long-range (allosteric) association between the α-rings and the catalytic sites.

Herein we report the results of our investigation on the conformational and functional effects on CP activity of 5-(phenyl)-10,15,20-(tri *N*-methyl-4-pyridyl) porphyrin (Tris-T4; Figure 2). In this compound, a phenyl group replaces a cationic *N*-methyl-4-pyridyl group, with the consequent loss of a positive charge on the porphyrin scaffold. Importantly, changing the ionic match with the 20S α-ring surface, and at the same time introducing a hydrophobic moiety in the structure, turns the activity of the compound from blocking (inhibitor) to lock picking (activator).

With the aim of elucidating the relation between 20S conformational transitions and the Tris-T4 effects on the hydrolytic activity, we investigated the interaction of Tris-T4 with h20S, wild type yeast (wt y20S), and the yeast α3ΔN mutant of 20S proteasome, a form characterized by the disruption of the gate closing mechanism [39,40]. We performed enzyme assays, kinetic analyses, NMR studies and in silico simulations addressing binding modes, conformational transitions and chymotriptic-like activity of Tris-T4/CP complexes.

## 2. Results

### 2.1. Modulation by Tris-T4 of the Steady-State Chymotryptic-Like Activity of 20S Proteasome

In a previous report [36], we compared the effect of a series of cationic porphyrins, including Tris-T4, toward the enzymatic activities of purified rabbit 20S proteasome, employing a 100 µM concentration of fluorogenic substrate. According to our previous SARs, which related the inhibitory potency to the number of positive charges present on the porphyrin structure, the apparent inhibitory effect of Tris-T4 was lower than that shown by the tetra-cationic porphyrin H2T4 at this substrate concentration [37]. Nevertheless, the value of the IC_50_ (0.92 ± 0.06 µM, chymotryptic-like activity) was such that a further investigation of the Tris-T4 inhibitory behavior appeared worthwhile.

In order to better characterize the effect of Tris-T4 on the functional properties of the 20S proteasome, we have carried out an investigation on the steady-state chymotryptic-like activity of h20S as a function of Tris-T4 concentration, employing different substrate concentrations.

**Figure 3:**
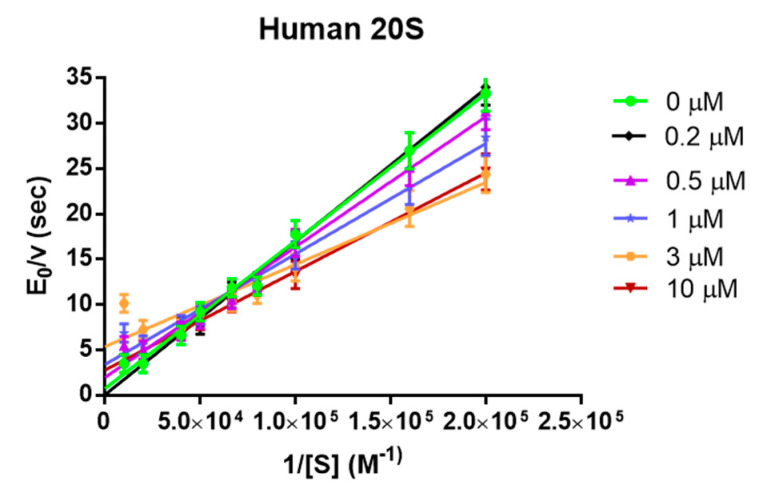
Lineweaver-Burk double reciprocal plot as a function of substrate concentration of 20S proteasome chymotryptic-like activity at different Tris-T4 concentrations, ranging from 0 to 10 μM. For the sake of clarity, not all Tris-T4 concentrations have been reported. Continuous lines are the non-linear least-squares fitting of data according to Equation (4).

Hereafter, the Lineweaver-Burk double-reciprocal plot is shown (Figure 3) as a function of the substrate concentration (between 5 µM and 100 µM) at different Tris-T4 concentrations spanning between 0 and 10 μM. It appears evident in Figure 3 that, at (Substrate) > 25 μM, the linearity of the double-reciprocal plot is lost, as an increasing substrate concentration brings about a slowing down of the enzymatic activity, as also reported by others [41]. This feature, which might underlie a structural change, induced by the substrate binding, is magnified as the concentration of Tris-T4 is enhanced. As a matter of fact, from Figure 3 it comes out that at (Substrate) < 25 µM Tris-T4 follows a Michaelis-Menten behavior and, unlike H2T4 [37], acts as an activator of the 20S proteasome.

Therefore, in previous observations at 100 µM substrate the strong activation effect, observed at lower substrate concentrations (see Figure 3), was hidden by this inhibitory effect at higher substrate concentrations. Consequently, we have limited our analysis according to Equation (4) (see experimental section) to a range of (Substrate) ≤ 25 μM.

From the qualitative standpoint, it becomes evident that upon addition of Tris-T4 the slope of the double-reciprocal plot decreases, envisaging an increase of k*_cat_*/K*_m_*, and thus of the chymotryptic-like enzymatic activity of h20S proteasome, accompanied by a decrease of both the K*_m_* and, to a lesser extent, the k*_cat_*.

**Figure 4:**
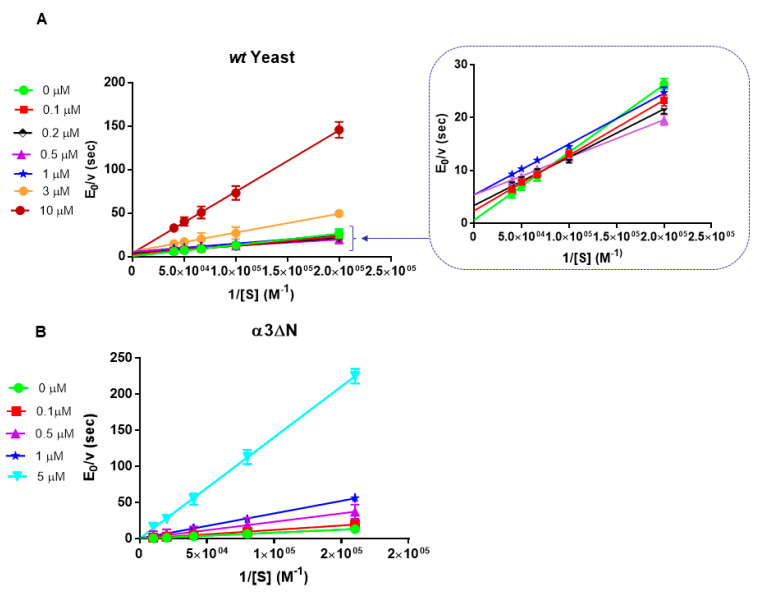
Lineweaver-Burk plot of the effect of Tris-T4 wt y20S proteasome (panel **A**) and on the yeast α3ΔN mutant (panel **B**). Various symbols refer to the different Tris-T4 concentrations, ranging from 0 to 10 μM (panel **A**) and from 0 to 5 μM (panel **B**). Inset refers to data for wt y20S at higher substrate concentrations to magnify the activation effect.

Then, we have extended the investigation on the functional modulation of the chymotryptic-like activity by Tris-T4 porphyrin also to wt y20S proteasome and to its mutant α3ΔN, which is characterized by a significant enhancement of the enzymatic activity, ascribed to disruption of the gate closing mechanism [39]. Figure 4 shows the Lineweaver–Burk plot for wt y20S (panel A) and for the α3ΔN mutant (panel B).

Several points emerge from this first step analysis, namely (i) the effect of Tris-T4 on the chymotryptic-like activity of h20S and wt y20S proteasomes is exerted through an activation, which is essentially completed at 1 μM Tris-T4 (for wt y20S, see Figure 4, panel A) and at 3 μM Tris-T4 (for h20S, see Figure 3); (ii) in the case of wt y20S at higher Tris-T4 concentrations a competitive inhibitory effect (never observed in h20S, at least over the investigated concentration range) overwhelms the activation, bringing about a drastic decrease of the enzymatic activity (Figure 4, panel A); (iii) in the case of the α3ΔN mutant only the competitive inhibitory effect is observed (Figure 4, panel B); (iv) the extent of substrate-linked inhibitory (investigated only for h20S proteasome) becomes more and more evident as Tris-T4 concentration is increased (see Figure 3).

**Figure 5:**
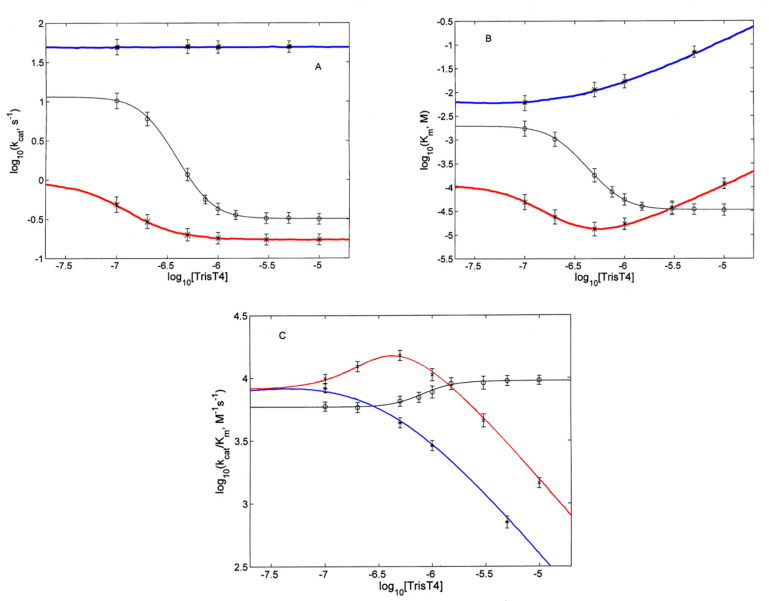
Dependence on Tris-T4 concentration for human (o; black line), wild type yeast (x; red line) and yeast α3ΔN mutant (*; blue line) 20S proteasome for k_cat_ (panel **A**), Km (panel **B**) and k_cat_/K_m_ (panel **C**). Continuous lines are obtained by non-linear least-squares fitting of data according to Equations (1)–(3), respectively, employing parameters reported in Table 1.

**Scheme 1:**
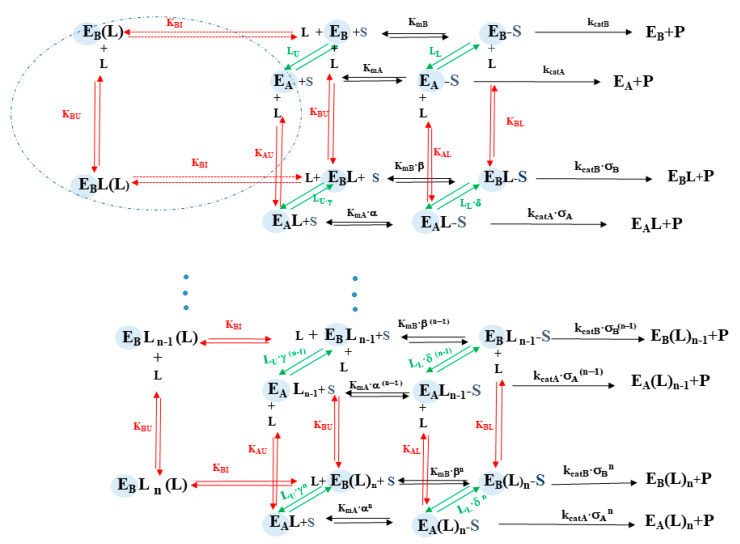
Upper part is the scheme for the first Tris-T4 binding step, while in the lower part is the scheme for the last *n*-th Tris-T4 binding step. The left part (enclosed in the blue circle) concerns the binding of Tris-T4 to the competitive inhibitory site, concerning only the substrate-free enzyme in the B state. Parameters concerning the enzymatic activity are depicted in black, those regarding porphyrin binding are depicted in red and conformational equilibria are depicted in green; S and L bound to E are indicated in larger font size. The meaning of various parameters is: k_catA_ and k_catB_ are the rates of the rate-limiting step in the two states A and B; K_mA_ and K_mB_ are the Michaelis-Menten constants in A and B; K_AU_ and K_BU_ are the affinity constant for Tris-T4 of the 20S proteasome in the absence of substrate in the two states A and B, K_AL_ and K_BL_ are the affinity constant for Tris-T4 of the 20S proteasome in the substrate-bound ES complex, while K_AI_ and K_BI_ are the affinity constant for Tris-T4 to the (eventual) inhibitory competitive site of the 20S proteasome. L_U_ is the equilibrium constant between A and B in the absence of the substrate, L_L_ is the equilibrium constant between A and B in the substrate-bound ES complex.

Altogether, these evidences suggest that Tris-T4 binding stabilizes a functional state, which is in allosteric equilibrium with that predominant in the free enzyme, appears characterized by a higher enzymatic activity and a slower rate-limiting step (i.e., k_cat_), as suggested by the behavior observed in h20S and wt y20S proteasome (see Figure 3, Figure 4A and Figure 5A). Further, the steep dependence on Tris-T4 concentration of measured catalytic parameters (i.e., k_cat_ and K_m_, see Figure 5A,B) indicates that this stabilization is a cooperative process, involving the concerted effect of more than one porphyrin molecule. This feature can be accounted for by a two-state allosteric model [42], implying the existence of a fast equilibrium between (at least) two conformational states (e.g., A and B, see Scheme 1). Both the substrate and Tris-T4 have a different affinity for the two states, their binding affecting the equilibrium to a different extent. In particular, as the Tris-T4 concentration rises up, the A state (which is prevalent in the absence of the substrate and of Tris-T4) is progressively destabilized in favor of the B state, which has a higher affinity for Tris-T4 and, to a lesser extent, for the substrate. It is important to point out that A and B functional states are general expressions of two structural arrangements and they might refer to the 20S “closed-gate” and “open-gate” conformations, often mentioned in the literature. Nevertheless, they can also be viewed as more local structural changes and/or more widespread conformational/functional ensembles, including “open” and “closed” sub-states, in which the functional properties are varied. The occurrence of a Tris-T4-linked conformational transition accounts very well for the steep dependence on Tris-T4 of the catalytic parameters for the chymotryptic-like activity, which appears very cooperative and restricted within a very limited range of concentrations (see Figure 5A–C).

The analysis of data reported in Figure 5 is based on the thermodynamic system, described above and sketched in Scheme 1. The meaning of the different parameters is described in the legend of Scheme 1, where E_A_ and E_B_ represent the unbound states, while E_A_·S and E_B_·S the substrate bound states. This Tris-T4-linked conformational shift occurs through a concerted mechanism, implying the binding of multiple Tris-T4 molecules, *n* being the interaction parameter, which reflects the minimum number of porphyrin units bound cooperatively to their site on 20S proteasome.

In addition, for the sake of clarity, in Scheme 1 some of these parameters are reported as interaction ratios, namely α = *K_AU_*/*K_AL_*, β = *K_BU_*/*K_BL_*, γ = *K_BU_*/*K_AU_*, and δ = *K_BL_*/*K_AL_*. Finally, σ_A_ and σ_B_ refer to the eventual effect of Tris-T4 binding on the catalytic rate-limiting step *k_cat_* of either state *A* and/or state *B* (see Scheme 1). Therefore, the dependence of catalytic parameters, measured from data reported in Figure 3 and Figure 4, is described by continuous lines in Figure 5A–C, and it has been carried out employing Equations (1)–(3) where *^obs^k_cat_*, *^obs^K_m_*, and *^obs^k_cat_*/*^obs^K_m_* are the observed catalytic parameter at a given concentration of Tris-T4, ^0^*K_m_* corresponds to *K_m_* in the absence of Tris-T4, *n* is the number of concerted allosteric binding sites for *Tris-T4*, and *m* is the number of competitive inhibitory sites.

(1) kobscat=kcatA⋅(1+KAL⋅[Tris_T4])n+LL⋅kcatB⋅(1+KBL⋅[Tris_T4])n(1+KAL⋅[Tris_T4])n+LL⋅(1+KBL⋅[Tris_T4])n

(2) Kobsm=K0m⋅(1+KAU⋅[Tris_T4])n+LU⋅(1+KBI⋅[Tris_T4])m⋅(1+KBU⋅[Tris_T4])n(1+KAL⋅[Tris_T4])n+LL⋅(1+KBL⋅[Tris_T4])n

(3) kobscatKobsm=kcatAKmA⋅(1+KAU⋅[Tris_T4])n+LU⋅kcatBKmB⋅(1+KBI⋅[Tris_T4])m⋅(1+KBU⋅[Tris_T4])n(1+KAU⋅[Tris_T4])n+LU⋅(1+KBI⋅[Tris_T4])m⋅(1+KBU⋅[Tris_T4])n

In the case of h20S proteasome, *n* = 3 and *m* = 0 turned out the minimum value to account for the quite steep dependence of catalytic parameters for h20S proteasome, as reported in Figure 5A–C. On the other hand, for y20S proteasome (both for wild type and α3ΔN mutant), the value was *n* = 2 and *m* = 1.

In this respect, it may be important to outline that, in the case of the wt y20S proteasome, a value of *n* > 2 cannot be employed for the description of the Tris-T4-linked effect on catalytic parameters (Figure 5). Table 1 reports the parameters for the substrate (i.e., *k_cat_* and *K_m_*) and Tris-T4 (i.e., *K_U_*, *K_L_* and *K_I_*) interactions with 20S proteasome in the two conformations A and B.

It is important to outline the high reliability of most parameters, reported in Table 1. First of all, as from Equation (1), the dependence of *k*_cat_ on Tris-T4 concentration (Figure 5A) allows to validate accurately values of *k_catA_*, *k_catB_*, *K_AL_* and *K_BL_*. These parameters are then kept fixed for the description of the Tris-T4-linked activation effect on *K_m_* (Figure 5B) and *k_cat_*/*K_m_* (Figure 5C), employing Equations (2) and (3); this allows the accurate validation of *K_mB_*, *K_AU_* and *K_BU_*. Finally, the inhibitory effect for y20S, which we have imposed to be effective only in the B conformation, characterizes univocally *K_BI_*. Additional constraints, which render parameters in Table 1 even more well defined, are (i) each one of affinity constants for Tris-T4 (i.e., *K_AU_*, *K_BU_*, *K_AL_*, and *K_BL_*) is fixed to the same value (for each one of the four states E*_A_*, E*_B_*, E_A_-S and E*_B_*-S, see Scheme 1) independently on the number of Tris-T4 bound molecules, as in a canonical two-state allosteric model [42], and (ii) *k_catA_* and *k_catB_* do not vary upon Tris-T4 binding (i.e., σ_A_ = σ_B_ = 1).

The only floating parameters are L*_U_* and L*_L_*, which is always true for a two-state allosteric model [42]. As already outlined before, wt *y*20S proteasome (though showing different catalytic parameters, see Table 1) displays a Tris-T4-linked behavior closely similar to that observed for h20S proteasome up to 1 μM Tris-T4 (as suggested by the very similar porphyrin binding parameters between the two 20S proteasomes, see Table 1), while at higher porphyrin concentrations a competitive inhibitory behavior appears (Figure 4, panel A). This result underlies the existence of an additional site for Tris-T4 in y20S proteasome, which becomes evident only upon the stabilization of the B state, by consequence, either the site is not present in the A state or its affinity is too low to be detected. In any case, we assumed K*_AI_* = 0. On the other hand, in the α3ΔN mutant no Tris-T4-linked activation effect is detected and only the competitive inhibitory effect is observed (Figure 4, panel B). On the basis of the parameters reported in Table 1, the apparent lack of activation in α3ΔN mutant is due to the very low free energy difference between the A and B functional states in the mutant (ΔG_A-B_ = RTln(L*_U_*), see L*_U_* in Table 1; see the Experimental section for details), such that already upon binding of the first Tris-T4 molecule the conformational equilibrium is strongly shifted in favor of the B functional state (see also Discussion). This peculiar behavior is likely referable to the gross alteration of the mechanism connecting gating dynamics to substrate hydrolysis due to the mutation, which drastically reduces the free energy for the A → B transition, thus hiding the activation process, so that α3ΔN mutant only displays the competitive inhibitory effect by the second Tris-T4 molecule. The close energy levels between A and B renders more complicated a characterization of the A functional state in the case of α3ΔN mutant, so that, for the sake of clarity, we have imposed the same value of k*_cat_* for the two states. On the other hand, the substrate affinity (K*_m_*) differs between A and B states of α3ΔN mutant as well as with respect to wt y20S, whereas the values of the binding constants of Tris-T4 remain the same in the wild type and the mutant (Table 1). The only exception concerns K*_AL_* which, however, is more difficult to characterize because of the lower thermodynamic stability of the A conformation in α3ΔN mutant. In any event, the close similarity for most of Tris-T4 binding parameters between wt y20S and α3ΔN mutant suggests that Tris-T4 binding sites are not significantly altered by the mutation characterizing α3ΔN structure, while, on the contrary, the native molecular mechanism leading from the ligand binding to the allosteric activation of the catalytic activity is deeply altered.

### 2.2. NMR Analysis of Tris-T4/20S Proteasome Complexes

For NMR interaction studies, Saturation Transfer Difference (STD) and WaterLogsy (WL) experiments [43,44] were employed to derive the ligand binding epitope.

As a starting point, the Tris-T4 structure has been analyzed by ^1^H NMR spectroscopy in the buffer (50 mM Hepes, 100 mM NaCl and 1mM DTT). The ^1^H chemical shift assignments of Tris-T4 are reported in Supplementary Figure S1. Both STD- and WL-NMR spectra of Tris-T4 were acquired in a 200-fold excess over the h20S proteasome [37,45].

**Figure 6:**
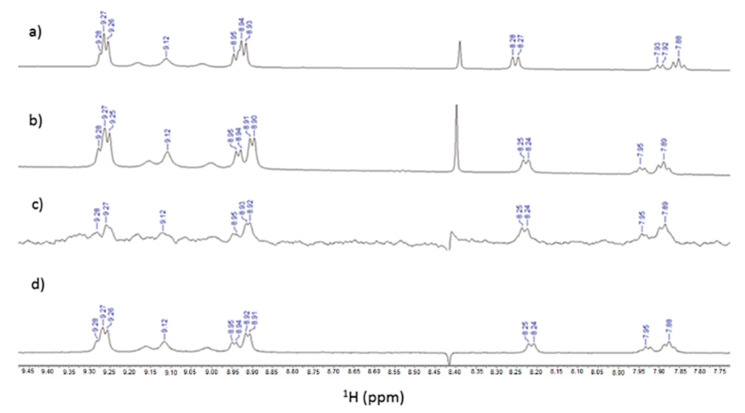
(**a**) Low-field of ^1^H NMR spectrum of Tris-T4 (175 μM) in the buffer (50 mM Hepes, 100 mM NaCl and 1mM DTT); (**b**) in the presence of a 20S proteasome (860 nM); (**c**) STD and (**d**) WaterLOGSY spectra.

The ^1^H NMR spectra of Tris-T4 in HEPES (50 mM), NaCl (100 mM) solution are reported either alone (Figure 6a) or in the presence of the h20S proteasome (Figure 6b).

The ^1^H Tris-T4 chemical shifts did not significantly change upon the addition of 20S proteasome, except for the phenyl ring resonances.

**Figure 7:**
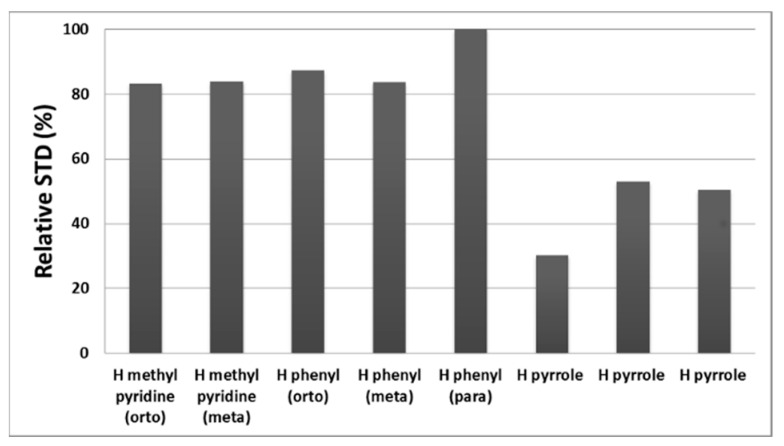
Relative intensity of STD signals of the individual protons for Tris-T4 normalized to that of the most intense not overlapped signal.

In particular, ^1^H phenyl meta and para hydrogens displayed soft chemical shift downfield perturbations (from 7.93 to 7.95 ppm and from 7.88 to 7.89, respectively) whereas the phenyl ortho hydrogen showed an up-fielded chemical shift (from 8.28 to 8.25 ppm). STD (Figure 6c) and WL-NMR spectra (Figure 6d) of Tris-T4 in the h20S proteasome clearly show positive signals; this result is thus indicative of Tris-T4/20S interaction. Then, to identify the ligand moiety more closely interacting with the protein, we evaluated and compared the saturation effects of the individual Tris-T4 proton resonances (I_STD_ = I_0_ − I_sat_) [46,47]. The signal showing the largest I_STD_/I_0_ value, the para phenyl proton, was normalized to 100% (Figure 7). The relative degree of saturation of the individual protons, normalized to that of the para phenyl proton, was then used to compare the STD effect.

As shown in Figure 7, the phenyl moiety together with methyl-pyridine rings are mainly involved in the 20S binding. On the other hand, the STD data indicate that the pyrrole ring is likely less directly involved in the recognition of the 20S proteasome.

As a control, a STD experiment with no added 20S was also carried out; under these conditions, no signals were detected in the STD spectrum, suggesting that the saturation in the presence of 20S proteasome does not originate from non-specific interactions.

Furthermore, to gain insight into the binding of Tris-T4 to proteasome, STD and WL experiments of Tris-T4 in the presence of α3ΔN y20S proteasome were acquired (Supplementary Figure S2) under the same condition used for Tris-T4 and h20S. The NMR spectra of Tris-T4 in the presence of α3ΔN y20S is consistent with the results obtained with Tris-T4 in the presence of h20S. Indeed, similar STD effects were observed for phenyl protons together with methyl-pyridine protons. Interestingly, a significant intensity reduction in the STD spectrum respect to those acquired with Tris-T4 and h20S was observed, indicating a smaller number of binding molecules, in agreement with the results of kinetic studies.

### 2.3. In Silico Studies

#### 2.3.1. Ligand Analysis

The tri-cationic form was calculated as the prevalent ionic form of Tris-T4 at cytoplasmic pH (7.2) (79%; Supplementary Table S1 and Figure S3). Accordingly, the Tris-T4 molecular model was built and subjected to conformational analysis by means of molecular dynamics calculations (simulated annealing, SA) combined with molecular mechanics (MM) energy minimization. Finally, the obtained minima were subjected to full geometry optimization by quantum mechanical method (MOPAC, PM7) (see the Experimental Section for details). The resulting conformers were analyzed and their pharmacophoric features were calculated based on the results of the NMR analysis of the Tris-T4/h20S complexes.

**Figure 8:**
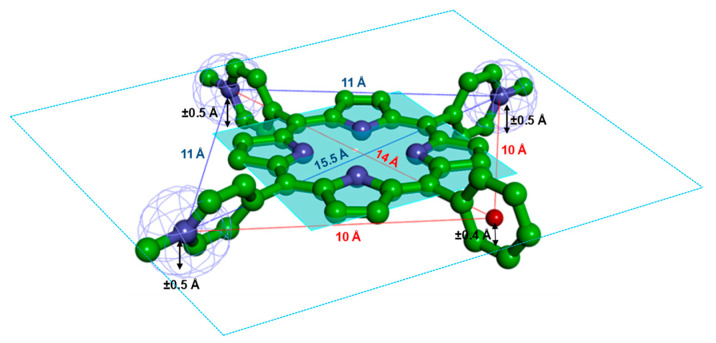
Tris-T4 pharmacophore and related inter-atomic distances and plane heights: (i) the positive ionizable pyridine nitrogen atoms are evidenced as mesh spheres; (ii) the centroid of the phenyl ring is displayed as a red ball and iii) the plane of the porphyrin ring is displayed and coloured in cyan. The global minimum conformer of Tris-T4 is displayed in ball&stick and coloured by atom type (C: green; N: blue).

All conformers showed a distance of ~11 Å between protonated nitrogen atoms of adjacent *N*-methyl pyridine rings, as also found for H2T4 (root mean square deviation (RMSD) value of pyridine nitrogen atoms = 0.07 Å). Since in Tris-T4 one pyridine moiety is replaced by a phenyl ring, a different set of interatomic distances between the charged groups is observed with respect to H2T4, resulting in an overall different pharmacophore containing a hydrophobic feature (Figure 8).

In the subsequent dynamic docking studies in complex with h20S, the Tris-T4 pharmacophore was employed for the generation of the binding hypothesis while the global minimum conformer was used as input ligand structure (see below).

#### 2.3.2. Docking Studies on the Tris-T4-h20S Interaction

Docking simulations were performed on both the closed and open forms of h20S proteasome by using the atomic molecular models previously developed by us [37] (template PDB structures: 4R3O and 5T0J for the closed and open state, respectively).

By fitting the Tris-T4 pharmacophore on h20S closed and open structures, we selected as putative binding sites those where (i) the positively charged methyl-pyridine rings could establish ionic interactions with h20S negatively charged residues (Supplementary Table S2), and, at the same time, (ii) the phenyl ring could be placed in a suitable hydrophobic pocket. The obtained complexes were used as starting structures in the subsequent molecular dynamics docking studies ^§^.

We initially simulated the binding of the first Tris-T4 molecule to both the closed and open forms of h20S. Using the closed h20S as starting structure, docking results showed the best ranked, hence selected, Tris-T4/h20S complex with the ligand bound at the α5–α6 groove (ligand RMSD with respect to the starting position = 10.89 Å) (Supplementary Figure S4; Table S4).

On the other hand, when a Tris-T4 molecule was docked to the open structure of h20S, the best docked complex showed Tris-T4 bound at the α4–α5 groove (ligand RMSD with respect to the starting position = 9.53 Å) (Supplementary Figure S5; Table S5). By comparing the interaction energy values of the best-ranked complexes (Supplementary Tables S4 and S5), the Tris-T4 molecule showed a more favorable binding energy when docked to the open h20S structure. Since it is known that substrate-bound 20S shifts to the open state [48,49], this finding reconciles with kinetic data, which indicated a higher affinity of Tris-T4 for the substrate-bound forms of h20S (Table 1).

**Figure 9:**
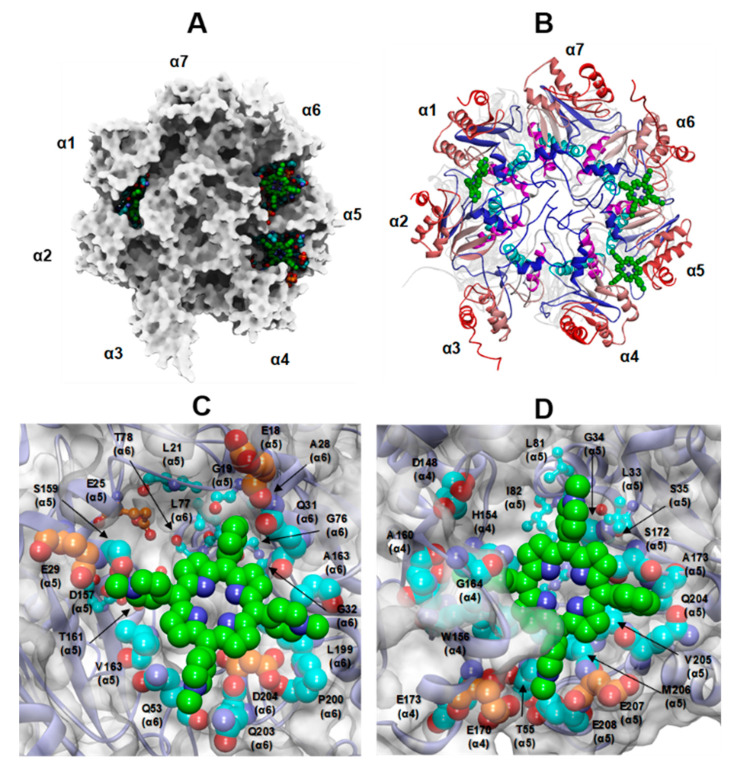
(**A**,**B**) Top view of the selected docked complex of three Tris-T4 molecules bound to human 20S (closed conformation). (**A**): the protein is displayed as Connolly surface and coloured in grey except for residues involved in interactions with Tris-T4, which are displayed as CPK. (**B**): the protein is displayed in ribbons and secondary structure elements of each subunit are sequentially coloured from N- to C-terminal end (αH0 = blue; αH1 = cyan; αH2 = magenta, αH3-αH5 = light red to dark red). C-D: Close up view on the ligand binding site at the α5–α6 (**C**) and α4–α5 (**D**) grooves. Tris-T4 is coloured by atom type (C: green; N: blue). The residues involved in interactions with Tris-T4 are displayed in CPK and coloured: orange (negative residues involved in ionic interactions with RPs) and cyan (residues not engaged in RP interactions). The residues involved in interactions with RPs hydrophobic motif are evidenced in ball&stick.

**Figure 10:**
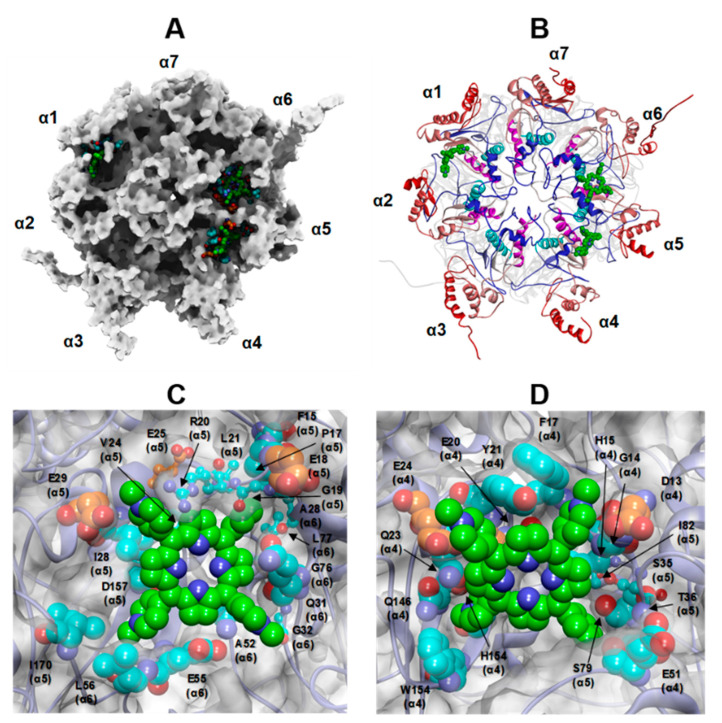
(**A**,**B**): Top view of the selected docked complex of three Tris-T4 molecules bound to human 20S (open conformation). (**A**): the protein is displayed as Connolly surface and coloured in grey except for residues involved in interactions with Tris-T4, which are displayed as CPK. (**B**): the protein is displayed in ribbons and secondary structure elements of each subunit are sequentially coloured from N- to C-terminal end (αH0 = blue; αH1 = cyan; αH2 = magenta, αH3–αH5 = light red to dark red). C-D: Close up view on the ligand-binding site at the α5–α6 (**C**) and α4–α5 (**D**) grooves. Tris-T4 is coloured by atom type (C: green; N: blue). The residues involved in interactions with Tris-T4 are displayed in CPK and coloured: orange (negative residues involved in ionic interactions with RPs) and cyan (residues not engaged in RP interactions). The residues involved in interactions with RPs hydrophobic motif are evidenced in ball&stick.

Based on the outcome of kinetic experiments Equations (1)–(3), we continued our docking simulations considering the binding of two additional molecules of Tris-T4. When we used the h20S closed conformation as starting structure, the docked complex which turned out to be the most favorable in terms of non-bonded interaction energies (Supplementary Table S6) showed the three molecules of Tris-T4 bound at α5–α6, α4–α5, and α1–α2 grooves (Figure 9; Supplementary Figure S6A). The ligand molecules are differently located as compared to their starting position (ligand atom RMSD: 1st Tris-T4 molecule (α5–α6) = 8.68 Å; 2nd Tris-T4 molecule (α4–α5) = 16.23 Å; 3rd Tris-T4 molecule (α1–α2) = 6.86 Å) and establishing ionic interactions with fourteen negatively charged protein residues (Supplementary Table S11). Similarly, when we started from the h20S open conformation, the best ranked complexes showed the three molecules of Tris-T4 bound at the level of α5–α6, α4–α5, and α1–α2 grooves (Figure 10; Supplementary Table S7 and Figure S6B; all atom RMSD with respect to the starting position: 1st Tris-T4 molecule (α5–α6) = 9.12 Å; 2nd Tris-T4 molecule (α4–α5) = 7.92 Å; 3rd Tris-T4 molecule (α1–α2) = 13.73 Å), interacting with eleven negatively charged residues (Supplementary Table S12).

Noteworthy, in both cases, about half of these residues are also engaged in the binding of h20S to endogenous RPs, essentially located at the α4/α5 and α5/α6 grooves (Figure 9 and Figure 10; Supplementary Figure S6; Tables S11 and S12). Even more importantly, the Tris-T4 molecules bound at the α4/α5 and α5/α6 grooves also interact with h20S hydrophobic residues engaged in the binding with the C-terminal tails of RPs (Supplementary Tables S11 and S12). They include a highly conserved Leu residue at the edge of the αH1 helix of the 20S α-subunits (colored in cyan in Figure 9B and Figure 10B), whose side chain contributes to a hydrophobic surface patch that binds C termini of all known RPs, [12,20].

It is worth doing some considerations taking into account the whole of the docking solutions generated by our dynamic simulations (Supplementary Figures S7–S10, Tables S9–S12). Firstly, in the best docked complex, starting from either the closed or the open h20S structure, the molecule bound at α1–α2 groove displays a non-planar conformation of the porphyrin ring (Supplementary Figure S6; Table S8), and, accordingly, no other docking solutions showed the ligand bound at this groove. This prompted us to question a high affinity binding of Tris-T4 at α1–α2, as it can be hypothesized, instead, at the α4–α5 and α5–α6 grooves. Secondly, almost half of the docking solutions starting from the open h20S structure presented one Tris-T4 molecule bound to the negatively charged α5-loop part of the α-annulus (aa 120–135; available only when the gate is open), suggesting a possible involvement of this sub-structure in the 20S-Tris-T4 interaction. Lastly, since our docking procedure generates each complex starting from the previously produced one, the results of docking simulations also revealed the ability of the ligand molecule to move down through the grooves toward the catalytic chamber (Supplementary Figures S7–S10).

The selected docking solutions were checked against the obtained NMR data. At this aim, we calculated the solvent accessible surface (SASA; H_2_O probe) of the Tris-T4 molecules docked to the three binding sites, both starting from the open and closed h20S conformations. As shown in Figure S11, the phenyl ring and, in particular, the para-phenyl hydrogen, resulted the most shielded from the solvent when bound to the protein, followed by the methyl-pyridine rings and, then, the pyrrole core, in good agreement with the STD effects observed in the NMR analysis (Figure 7). To further investigate this issue, we calculated the rate of SASA decrease for the phenyl and *N*-methyl-pyridyl hydrogen atoms in our docking solutions with respect to their SASA in unbound Tris-T4 (Supplementary Table S13). The overall resulting trend reflects that reported for the observed STD effects, however, the docked complex obtained starting from the open h20S conformation evidenced the best fit to the NMR data with the para-hydrogen atom of the phenyl ring showing a 100% of SASA reduction in three putative binding sites.

#### 2.3.3. Analysis of the Tris-T4-Induced h20S Conformational Changes

In order to analyse the overall human 20S conformational variability during docking calculations (including SA) in complex with Tris-T4, firstly, a structural analysis was performed by means of atomic position deviations (root mean square deviation; RMSD) calculated on Cα atoms of the α and β rings. In particular, all complex structures obtained from docking studies (starting from both the 20S open and closed states and using either a single or three ligand molecules) were collected and analysed in order to underline common traits of intrinsic structural variability and/or dynamical features that may be associated to the binding events (Supplementary Figure S12). In this framework, atomic position deviations with respect to the starting conformations reached a comparable trend, spanning values from 0.54 to 0.66 nm (closed) and from 0.52 to 0.70 nm (open), with a RMSD mean value of the atomic position deviations for the whole set of structures of 0.58 nm. To explore the ability of Tris-T4 in modulating the closed/open conformational equilibrium of human 20S, we computed the RMSD using also the opposite starting structure as template (Supplementary Figure S12). This analysis evidenced that the ligand docked at the proteasome in the closed state (regardless of the specific binding site and the number of docked molecules) stabilizes a set of 20S conformations other than the starting closed state but even farther from the open state. On the other hand, Tris-T4 docked at the proteasome in the open state induced structural rearrangements across the protein pushing it toward an “intermediate” state between the open and closed conformations (comparable RMSD values with respect to open and closed proteasome starting structures; Supplementary Figure S12).

Then, to gain some clues on the structural details of Tris-T4 induced h20S conformation, we investigated the selected docked structures of Tris-T4 to h20S. Ligand-induced protein conformational changes were checked for their compatibility with the observed Tris-T4 induced shift toward a new h20S functional state characterized by enhanced substrate affinity (*K_m_*) and a slower rate-limiting step (k*_cat_*) (namely the B state; Table 1).

**Figure 11:**
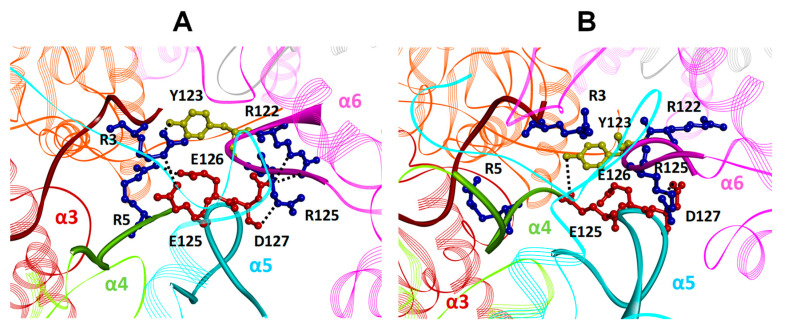
Close up view of the h20S α5-loop interaction pattern in (**A**) h20S closed conformation (PDB ID: 4R3O) and (**B**) h20S docked with three Tris-T4 molecules (starting from the closed conformation). The α subunits are rendered in cartoons and colored in pink (α1), orange (α2), brown (α3), light green (α4), cyan (α5), magenta (α6), and gray (α7). The α5-loop (cyan; aa120-135), the α6-loop (magenta; aa120-128), the α3 N-terminal tail (brown; aa1-15) and the α4 N-terminal tail (light green; aa1-13) are evidenced as solid ribbons. Key residues are displayed in ball&stick and colored in red (E125, E126 and D127 (α5)), blue (R122 and R125 (α6)) and yellow (Y123 (α6)).

It has been widely demonstrated that substrate affinity is increased by shifting the 20S conformational equilibrium toward the opening of the gate [22,48,49]. Thus, we analyzed the effect of Tris-T4 binding to the α-grooves on the closed gate conformation. The closing of the h20S substrate gate is granted by ionic interactions involving several charged residues. In particular, two arginine residues at the N-termini of the α3 (R3) and α4 (R5) interact with two consecutive glutamate residues (E125 and E126) located on the α5-loop (aa 120–135), making part of the substrate access pore (α-annulus) (Figure 11A).

This is accompanied by the formation of an H-bond involving a Tyr residue on the α6-loop of the α-annulus (Y123) and the α3 N-terminal tail (R3). Finally, the α5-loop and the α6-loop are coupled by an ionic interaction between D127 (α5) and R122 (α6) assisted by H-bonds between the side chain of R125 (α6) and the backbone of D127 (α5). In our docked complexes, the binding of Tris-T4 to the closed structure of h20S firstly occurs at α5–α6 groove (between the αH0 helices of α5 and α6; Figure 9 and Supplementary Figure S4) causing the breaking of the ionic interaction between the N-terminal tail of α4 (R5) and the α5-loop (E126) as well as of that between the negatively charged D127 of the α5-loop and the positively charged R122 of the α6-loop. Similarly, the binding of the second molecule at the α4–α5 groove pushes the αH0 helix of the α4 subunit toward the adjacent αH0 helix of α3 (Figure 9 vs. Supplementary Figure S13C), and, by consequence, further moves the α4 and the α3 N-terminal tails. This ultimately leads to the disruption of the ionic interactions between the N-termini of α4 (R5) and α3 (R3) and the α5- and the α6-loops (Figure 11A vs. Figure 11B).

The above described calculated perturbation of gate sub-structures is in line with the increased substrate affinity (*K_m_*) of the B functional state observed in human and yeast 20S (Table 1) as well as to the dramatic reduction of the free Gibbs energy difference between the A and B states in α3ΔN mutant (ΔG_A-B_ = RTln(L*_U_*), see L*_U_* in Table 1). Indeed, in the case of α3ΔN, the mutation prevents gate closing and, at the same time, disrupts the allosteric pathway connecting gating dynamics and substrate translocation and hydrolysis [39]. The breaking of the gate closure mechanism impairs the “open” to “closed” conformational transition, leading to a higher turn-over rate (higher k*_cat_*) of α3ΔN y20S mutant with respect to the wt y20S. However, the substrate affinity (*K_m_*) of α3ΔN mutant is decreased, as compared to wt y20S (see Table 1). To explain this apparent incongruence, we performed a structural analysis on the experimentally determined structure of α3ΔN *y*20S (PDB ID: 1G0U) [39] which revealed the replacement (i) of thirteen residue (121–133) of the key α5-loop with four Ala residues and (ii) of Y123 of the α6-loop with an Ala residue (Supplementary Figures S14 and S15; Table S14). The α5-loop and the corresponding loop of the α6 subunit are part of the substrate pore of 20S, regulating the translocation of substrates to the β5 catalytic center [27]. In the available experimentally determined structures, the opening of the 20S gate is matched with the “release” toward the pore cavity of either the α6-loop and the long and negatively charged α5-loop (Supplementary Figure S4C vs. Figure S5C) which is, accordingly, unstructured (i.e., not solved) in all 20S open structures.

In the E_D1-2_ states of h26S (PDB ID: 6MSJ and 6MSK), the two consecutive conformational states with the open gate conformation of 20S [12], the substrate is represented by the N-terminal tail of the protein chain descending from the above 19S pore cavity, the 20S closest residue (7.2 Å) being Y123 of the α6-loop.It can be hypothesized that during the subsequent (not solved) substrate translocation, the incoming N-terminal tail of the substrate is attracted by the negatively charged residues on the α5 loop to the below β5 catalytic chamber.

It has been reported that in the latent 20S proteasome, the substrate can be also processed accessing the gate from the C-terminal tail [50], in this case, it could be recruited directly by the α6-loop, thus approaching the β5 catalytic threonine from the opposite side.

On these bases, the presence of the α5 and α6 loops mutations in the α3ΔN y20S mutant (Supplementary Figures S14 and S15; Table S14) can account for the observed lower substrate affinity compared to the wild type despite the impairment of the substrate gate closing mechanism. The seven cryo-EM structures of 26S (i.e., h20S in complex with the RP 19S; 2.8–3.6 Å resolution) [12] captured during substrate unfolding and translocation and including 20S closed and open conformations, show that, as hypothesized for Tris-T4, also the RP 19S induce gate opening without directly contacting gate residues; instead, opening is accomplished via the long-range coupling between the 19S binding site and the mechanically key region of the 20S gate (i.e., allostery). In particular, the 19S Tyr residues at the Rpt1 (Y434) and Rpt5 (Y436) insert between the αH0 helices of adjacent 20S α-subunits (colored in blue in Figure 9B and Figure 10B). In the h26S structures with open h20S (E_D1_ and E_D2_ states; PDB ID: 6MSJ and 6MSK), these residues assume a specific orientation contacting the conserved Leu residues at α5 (L81) and α6 (L77), respectively. Importantly, Ruschak et al. proved that these conserved Leu residues are key elements in the allosteric network leading to *Thermoplasma acidophilum* 20S activation [20] (all the residues involved in the network identified by Ruschak et al. are evidenced in Figure 13). Thus, in order to challenge our Tris-T4 binding hypothesis, we compared the experimentally determined structures with the results obtained from our docking studies.

**Figure 12:**
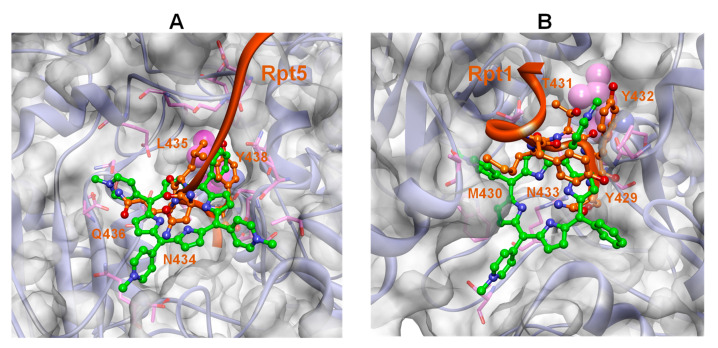
Structural overlap of the 19S C-terminal tails (i.e., Rpt5 and Rpt1) with Tris-T4 obtained by fitting the Cα atoms of the 20S α-ring of the Tris-T4/h20S best-docked complex (three ligand molecules; h20S starting conformation: closed) on the Cα atoms of the 20S α-ring of the h26S structure (ED_2_, open state; PDB ID: 6MSK). (**A**) Tris-T4 bound at the α5–α6 groove (green) and the Rpt5 C-terminal (orange) of 19S. (**B**) Tris-T4 bound at the α4–α5 groove (green) and the Rpt1 C-terminus (orange). The Rpt C-terminal residues overlapping with Tris-T4 are displayed in ball&stick and labelled. For sake of clarity, only the h20S structure of the docked complex is displayed. Residues interacting with both Tris-T4 and the Rpt residues are displayed in stick and coloured in pink. The vdW volume of L77 (α6; A) and L81 (α5; B) is shown in magenta.

As evidenced in Figure 12, the superimposition (by the h20S Cα atoms) of the best docked Tris-T4-h20S complex on the E_D1_ and E_D2_ states of h26S (PDB ID: 6MSJ and 6MSK), revealed a striking ability of the Tris-T4 molecules bound at the α4–α5 and α5–α6 grooves to reproduce the positioning of the side chain of the C-terminal tyrosine residues of Rpt1 and Rpt5 (HbYX activating motif), respectively, as well as their interaction with the key Leu residues on the αH1 helices (Supplementary Tables S11 and S12). Similarly, the structural comparison with the PA200-h20S X-ray complex (PDB ID: 6KWY) evidenced the ability of Tris-T4 to mimic the Tyr residues at the C-terminal tails of PA200 (Figure S16; Tables S11 and S12). These results support the hypothesis that Tris-T4, by binding to the α-grooves of the closed h20S structure is able to activate 20S exploiting the RP interaction sites.

**Figure 13:**
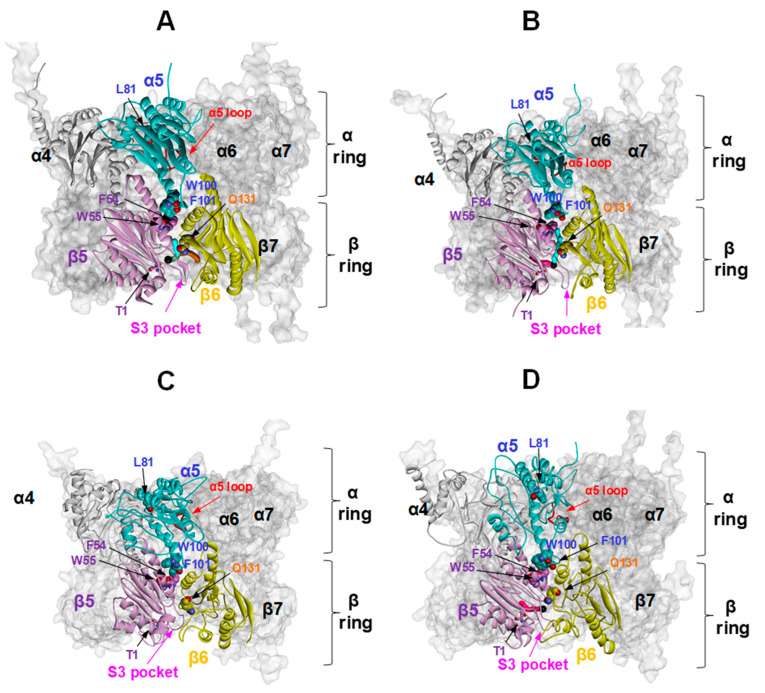
h20S proteasome tunnels at the catalytic β5-Thr1. α4/α5 and β5/β6 are shown in the foreground to focus on the tunnels generated around β5-Thr1 in closed (**A**) and open (**B**) 20S state and upon binding of three Tris-T4 molecules starting from the closed (**C**) and open (**D**) 20S state. α4/α5 and β5/β6 are rendered in solid cartoons (α4 = grey, α5 = cyan, β5 = pink, and β6 = green) whereas the whole body of the 20S proteasome is displayed in transparent surface; α5-loop is shown in red cartoons. A black solid ball evidences starting tunnel position; the tunnels are coloured in cyan, brown, and magenta. Catalytic Thr (T1) and key amino acids involved in the allosteric network described by Ruschak et al. [20] are labelled and shown in CPK with the same color of the corresponding subunit.

Then, we explored the long-range effects due to the binding of Tris-T4 molecules on the presence of solvent accessible internal tunnels leading from the catalytic threonine (T1), located at the N-terminal of the β5 (chymotryptic) catalytic subunit, to the bulk solvent. Indeed, the B state of both h20S and y20S, induced upon Tris-T4 binding, showed a decreased k*_cat_* (Table 1), which, according to the results of the kinetic experiments, is supposed to be due to a slower release of the products of the catalysis. Figure 13 reports the results obtained for the starting (open and closed) h20S structures compared to those obtained for the resulting Tris-T4 docked complexes. As it can be noted, different tunnels are found around the β5 catalytic T1 in the human 20S starting structures, depending on whether the enzyme is in the closed (Figure 13A) or open (Figure 13B) state. In particular, in the closed structure, a tunnel resulted at the β5-β6 interface, which bifurcates creating two exit points towards the solvent; the first one protrudes above the substrate (S3) pocket toward the exterior of the protein (colored in cyan in Figure 13A), whereas the other one leads to the 20S internal pore (colored in brown in Figure 13A). On the other hand, the h20S open state displays two different tunnels starting from the catalytic T1 residue of β5. One goes to the exterior of the protein, longer and partially overlapping with that present in the closed state (colored in cyan in Figure 13B), while the second one crosses the β5 subunit toward the β4 subunit and leads to the internal pore cavity (colored in magenta in Figure 13B).

The docking of Tris-T4 to h20S hampers the presence of tunnels connecting the β5 catalytic chamber to the bulk solvent (Figure 13C,D). In particular, in the closed structure the binding of one Tris-T4 molecule still allows the presence of a small tunnel next to the catalytic Thr and partially corresponding to that present in the open 20S state (data not shown), whereas in the presence of three ligand molecules the conformation of the S3 loop is changed so that the formation of tunnels or cavities is prevented (Figure 13C). On the other hand, in the open h20S structure upon the binding of one or three Tris-T4 molecule only the tunnel in proximity of the β4-β5 interface is preserved (colored in magenta in Figure 13B,D). In summary, the Tris-T4-induced h20S conformational changes resulting from our docking studies highlight a possible molecular mechanism by which Tris-T4 binding could on one hand prompt gate opening thus increasing substrate affinity (K*_m_*) but on the other hand impair substrate release, thus negatively affecting the turnover rate (k*_cat_*), in line with our experimental observations (Table 1).

## 3. Discussion

The existence of multiple proteasome conformations is a well-documented information, as shown by several authors [8,9,26,51]. The interaction of Tris-T4 with 20S proteasome clearly indicates that the functional modulation of 20S can only be accounted for by an allosteric equilibrium between (at least) two conformational/functional states, which we call A and B in general terms. As already mentioned, although these two functional states can be referred to the so-called 20S “closed” and “open” conformations, they do not mean to be bound to this specific structural change, the transition between them reflecting either a local and/or a more widespread conformational shift. Our binding hypothesis indicates the Tris-T4 cooperative interaction with key residues at the grooves present on the α-surface of h20S (α4–α5, α5–α6, and α1–α2) as responsible for the observed functional effects, with the phenyl moiety playing a key role in the molecular recognition event.

Functional characterization of the Tris-T4-linked effect on 20S has been carried out to a higher detail on h20S, but an analogous investigation has been undertaken also for wt *y*20S, in order to compare this effect between the two eukaryotic proteasomes.

Further, the study has been extended to α3ΔN *y*20S mutant, since in this way we were able to have a hint on the Tris-T4-induced effect also when the 20S substrate gate opening/closing mechanism is grossly altered [39]. From the functional viewpoint, a closer look at Scheme 1 and Table 1 puts in evidence that, in the absence of both substrate and Tris-T4, in h20S proteasome, the -ΔG^0^ of the A → B conformational transition equilibrium is 32.7 kJ/mol (ΔG_A0-B0_ in Figure S17).

In the substrate-bound state, the value of -ΔG^0^ for the AS → BS transition is 26.1 kJ/mol (corresponding to RTlnL_L_, see Table 1; ΔG_AS0-BS0_ in Figure S17), slightly decreased (by 6.6 kJ/mol) with respect to the unbound state. Therefore, in the absence of any additional factor, the observed binding and enzymatic processing of the synthetic substrate is still almost completely referable to the A functional state (circled area in Figure S17). This consideration is somehow supported by the evidence that h20S proteasome displays a Michaelis-Menten behavior, suggesting that the substrate alone is unable to induce the A → B conformational transition.

The molecular docking of the first Tris-T4 molecule to h20S displays different putative binding sites in the “closed” and “open” forms, the α5–α6 groove and the α4–α5 groove, respectively, showing a higher binding energy when bound to the “open” h20S. Kinetic data indicate a higher affinity of Tris-T4 for the h20S “open” substrate-bound form of both A and, at a higher extent, B functional states (K*_AL_*/K*_AU_* and K*_BL_*/K*_BU_*_,_ respectively; Table 1). In addition, the binding of additional Tris-T4 molecules either to the “closed” or to the “open” h20S conformation stabilizes the B functional state, consistent with the much higher affinity of this latter for Tris-T4 compared to the A state (about 4000-fold, K*_BU_*/K*_AU_* and K*_BL_*/K*_AL_*; Table 1).

As illustrated by the steep porphyrin concentration dependence of catalytic parameters (see Figure 5), it comes out a cooperative mechanism, involving the binding of (at least) three Tris-T4 molecules. The porphyrin binding brings about a conformational shift, such that in the substrate-free porphyrin triple-bound enzyme, the free energy for the A → B transition is 31.4 kJ/mol in favor of the B functional state (corresponding to RTlnL_U_γ^3^; ΔG_A3-B3_ in Figure S17). A comparable energy gain in favor of the B state is attained for the h20S substrate-bound conformation (corresponding to RTlnL_L_γ^3^; ΔG_AS3-BS3_ in Figure S17). Therefore, catalytic parameters for the chymotryptic-like synthetic substrate under saturating conditions of Tris-T4 (see Figure 3) essentially correspond to those characteristics of the B functional state, not yet described in literature. In particular, kinetic data indicate that each Tris-T4 binding step contributes by 21.4 kJ/mol (corresponding to RTln(K*_BU_*/K*_AU_*)) to bypass the free energy difference between the A and B functional states in the “closed” substrate-free conformation, and by 23.9 kJ/mol (corresponding to RTln(K*_BL_*/K*_AL_*)) in the “open” substrate-bound state (Figure S17).

The increasing destabilization of A state toward the B one facilitates the interaction of the porphyrin with the binding sites (characterized by K*_BU_*) further stabilizing the B state. Molecular docking simulations have allowed to propose that the Tris-T4-induced h20S conformational changes lead to: (i) the disruption of the “ionic lock” responsible for gate closing; (ii) the release of the charged residues of the α-annulus toward the pore channel (Figure 11), favoring the access of the substrate to the underlying chymotryptic site of the β5; (iii) the closing of most of the tunnels leading from the catalytic T1 to the bulk solvent (Figure 13).

Consistently, the B functional state of both h20S and y20S, induced upon Tris-T4 binding, shows an increased substrate affinity (*K_m_*; Table 1) and a decreased k*_cat_* (Table 1), which is supposed to be due to a slower release of the products of the catalysis.

Therefore, the slower k*_cat_* of the B functional state can be structurally explained as an increase of the energy barrier for the product release, due to the closure of some, though not all, exit pathways: this might also facilitate the substrate inhibitory behavior, as observed in Figure 3, giving rise to the apparent inhibitory effect of Tris-T4 at high substrate concentrations. Thus, the concerted h20S structural change induced by the binding of three porphyrin molecules, obtained by molecular docking simulations, is perfectly compatible with the allosteric mechanism observed in the functional data (Figure 5).

In the frame of the SARs of the previously developed porphyrin based analogues [36,37,38], the observed h20S activation by the cationic porphyrin Tris-T4 is due to the presence in the structure of a phenyl ring in place of the positively charged *N*-methyl-4-pyridyl moiety of the competitive inhibitor H2T4. Intriguingly, similarly to what experienced by us switching from H2T4 to Tris-T4 structure, it has been recently reported that the introduction of the hydrophobic motif HbYX in cationic (PR) peptides acting as 20S inhibitors, led to an activation of the ChT-L activity at low concentrations of the newly designed peptides [52]. The comparison of the Tris-T4/h20S docked complex with the experimentally derived structure of 26S, revealed a striking similarity between the binding mode of the Rpt C-terminal tails and the Tris-T4 molecules, with the Tris-T4 phenyl moiety reproducing the position and interactions of the key Tyr residue, including that of the conserved HbYX motif (Figure 12). Interestingly, STD and WaterLOGSY measurements put in evidence a major involvement of the Tris-T4 para phenyl proton in the interaction with the protein (largest I_STD_/I_0_ value; Figure 7), followed by the other phenyl protons and the methyl-pyridine protons, and, then, by the protons of the pyrrole core. Accordingly, docking results showed that the methyl-pyridine rings establish ionic interactions with the negatively charged residues involved in the binding of h20S with RPs, and, at the same time, the phenyl ring interacts with hydrophobic residues playing a key role in 20S activation (Figure 9, Figure 10 and Figure 12; Supplementary Tables S11 and S12). Furthermore, analysis of the solvent accessible surface area of Tris-T4 docked to h20S α-grooves, shows that, in line with the results of the NMR experiments, the phenyl ring of the complexed Tris-T4 is more embedded than the three pyridine rings, and both are more embedded than the pyrrole core (Supplementary Figure S11); however, only in the open conformation the para phenyl proton results the most embedded (Supplementary Table S13). The presence, either in the STD and WaterLOGSY experiments, of the para phenyl proton as the strongest signal supports, therefore, the hypothesis that Tris-T4 binding induces h20S to convert to the open gate conformation, from which is finally released within the equilibrium with free Tris-T4.

Taken together our kinetic, NMR, and computational results, highlight a possible scenario where the binding of Tris-T4 to the h20S α4–α5, α5–α6, and α1–α2 grooves (potentially at each of the two protein faces) involves the negatively charged residues interacting with the 19S, PA200, and PA28 RPs, together with the Leu residue at the edge of the αH1 helix, reported to be involved in 20S activation [20]. Thus, on one hand, the hydrophobic interaction stabilizes (h20S open state) or induces (h20S closed state) the opening of the gate favoring substrate recruitment (thus increasing substrate affinity), but on the other hand the ionic interactions “stick together” the adjacent α-subunits, thus limiting the 20S conformational freedom and leading to a lower turn-over rate, reflecting the calculated closure of the tunnel from the catalytic threonine to the exterior of the protein.

A comparison of parameters between h20S proteasome and wt y20S proteasome shows that, beside a difference for specific catalytic parameters (i.e., k*_cat_* and K*_m_*, see Table 1), the Tris-T4-linked activation mechanism is essentially the same. The similar pattern of conformations/functional states observed in human and in wt y20S proteasome (see Scheme 1) strengthens the relevance of the A/B conformational transition, which seems to be a highly conserved feature of proteasome among different organisms. Moreover, very similar values for the binding of Tris-T4 are observed in h20S and wt y20S with the only exception of the equilibrium constant for porphyrin binding to the free enzyme in the A functional state (i.e., K*_AU_*, see Table 1), which shows a higher affinity in wt y20S. Nevertheless, unlike h20S proteasome, wt y20S displays also an inhibitory competitive binding site for the porphyrin, which shows up only in the B functional state (Table 1). In other words, the B structure stabilization upon binding of Tris-T4 brings about in wt y20S the appearance of a high affinity site for the porphyrin, not observed in h20S, which exerts an inhibitory effect, nullifying the activation process due to the Tris-T4-linked conformational transition (Figure 4A). This effect becomes evident from the porphyrin-dependent behavior displayed by catalytic parameters (Figure 5), since after an initial enhancement of the turnover rate (i.e., k*_cat_*/K*_m_*, see Figure 5C), the stabilization of the B functional state leads to a marked inhibitory effect. This is mirrored by an analogous effect on K*_m_* (Figure 5B), such that after an initial decrease (corresponding to a higher substrate affinity) we observe a dramatic increase of K*_m_* consequent to the competitive inhibitory effect.

Our finding that the Tris-T4-linked activation of 20S proteasome affects the molecular mechanism responsible for gate opening and substrate recruitment is consistent with the results obtained for the yeast α3ΔN mutant, where this molecular mechanism is altered due to the mutation in the amino acid composition of the *α*-annulus region (Supplementary Figures S14 and S15). Indeed, in the *y*20S α3ΔN the gross alteration to the gating mechanism contributes to the deeply reduction of the energy difference between the A and B functional states (8.3 kJ/mol; Supplementary Figure S18). As a consequence, the competitive inhibitory effect of Tris-T4 dominates the behavior of *y*20S α3ΔN mutant (Figure 4B). Since the 20S/Tris-T4 affinity constants, in line with our binding hypothesis, do not seem to be affected in yeast α3ΔN mutant with respect to wt proteasome (Table 1), then, the binding of a single molecule of Tris-T4 is sufficient to stabilize the B functional state (Supplementary Figure S18) and no activation is observed (see Figure 4B and Figure 5). In line with the outcomes of functional studies, the putative Tris-T4 binding sites are conserved among h20S, *y*20s and *y*20S α3ΔN mutant (Supplementary Tables S11 and S12; Figure S15), while, at the same time, the proposed allosteric mechanism for the observed Tris-T4 effects could not be reproduced in *y*20S α3ΔN due to the lack of key functional residues. Finally yet importantly, the NMR experiments have confirmed the interaction between Tris-T4 and α3ΔN y20S proteasome, showing that Tris-T4 interacts with α3ΔN y20S similarly to what was observed with h20S and likely with a smaller number of molecules, since the signal to noise ratio is significantly lower (Supplementary Figure S2).

## 4. Materials and Methods

### 4.1. Chemicals

Purified human 20S proteasome and the fluorogenic substrate Suc-LLVY-AMC, used to test the ChT-L, were purchased from Boston Biochem (Cambridge, MA, USA). Tris-T4 5-(phenyl)-10,15,20-(tri *N*-methyl-4-pyridyl) porphyrin was purchased from Mid-Century Chemicals. Yeast CP 20S wild-type and the mutant form, α3ΔN, were purified as previously reported [39].

### 4.2. 20S Proteasomal Enzyme Assays and Analysis of Kinetic Data

20S proteasome activity assays were performed in vitro in the assay buffer (i.e., 50 mM TrisHCl, pH 8.0) by mixing 20S proteasome (2 nM) with increasing concentrations (0.1–10 µM) of Tris-T4 incubating for 30 min at 37 °C. Then, varying concentrations (between 5 µM and 100 µM) of fluorogenic peptide were added in a 384 multiwell black plate. A minimum of three replicates were performed for each data point. The released AMC fluorescence was recorded at 440 nm (excitation at 360 nm) for 45 min, which is a time interval over which linearity was observed in a fluorescence plate reader (Varioskan, Thermo). Data relative to CP activities at different substrate concentrations have been analyzed by a double reciprocal Lineweaver–Burk plot, according to the following equation:

(4) [E0]v=Km±kcatx 1[S]+1kcat

where (*E*_0_) is the enzyme concentration, *ν* is the observed velocity (expressed as moles of substrate cleaved per time interval unit), (*S*) is the substrate concentration, *K_m_* is the Michaelis–Menten equilibrium constant, referring to the affinity for substrate, and *k*_cat_ is the velocity of the rate-limiting step. All curve fitting and statistical analysis were carried out using the non-linear fitting tool (NLFit) and MatLab (The Math works Inc., Natick, MA, USA). The parametric data fitting was based on nonlinear regression and the method of least squares. Model discrimination and choice was based on the goodness of fit. The goodness of fit was evaluated by visual examination of the fitted curves, 95% confidence bounds for the fitted coefficients and statistical analysis for determining the square of the multiple correlation coefficient (*R*^2^).The Gibbs free energy difference (ΔG^0^ = −RT lnK) was calculated using the values of the equilibrium constants reported in Table 1 and corresponds to −ΔG^0^ of the considered conformational transition equilibrium as depicted in Scheme 1.

### 4.3. NMR Measurements

The complete experimental description of NMR studies is reported in the Supplementary Materials.

### 4.4. Molecular Modelling

Molecular modelling calculations were performed on SGI Origin 200 8XR12000 and E4 Server Twin 2 × Dual Xeon 5520, equipped with two nodes. Each node: 2 × Intel Xeon QuadCore E5520, 2.26 Ghz, 36 GB RAM. The molecular modelling graphics were carried out on a personal computer equipped with Intel(R) Core (TM) i7-4790 processor and SGI Octane 2 workstations. The complete experimental description of bioinformatics and molecular modelling studies is fully detailed in the Supplementary Materials.

## 5. Conclusions

In conclusion, we have put in evidence that an uncharacterized less abundant functional state of h20S proteasome, referred as B, can be stabilized by the binding of the porphyrin-based ligand Tris-T4, leading to the increase of the ChT-L catalytic activity. Namely, we characterized the equilibrium between two h20S functional states, A and B, each one being composed of a manifold distribution of sub-states. In the absence of any ligand the A functional state is largely prevalent, and it remains predominant even in the presence of a synthetic fluorogenic substrate. On the contrary, the binding of Tris-T4 is able to shift, even in the absence of the substrate, the equilibrium in favour of the B functional state, which is characterized by a much higher substrate affinity and a higher catalytic efficiency. According to our binding hypothesis, the h20S activation observed upon Tris-T4 binding and described in this paper might simulate to some extent the allosteric activation by regulatory proteins and/or peptides and a possible common pharmacophore has been identified. Indeed, our Tris-T4/h20S molecular interaction model proposes partially overlapping binding sites with RPs for the cooperative binding of Tris-T4 to h20S, with the phenyl moiety playing the role of the key Tyr residue present at the Rpt C-terminal tails (including the conserved HbYX motif). It is worth recalling that porphyrins already find therapeutic application in photodynamic therapy for the treatment of oncological and ophthalmological pathologies. Thus, the functionalization of porphyrin derivatives to target proteasome may bring about a wider repertoire of clinical applications.

Although an exhaustive description of all the conformations explored by proteasome during its function is challenging and beyond the aim of this report, we believe that our results opens a novel scenario in the description of the proteasome modulation mechanism by exogenous factors, paving the way for the design of a second generation of h20S allosteric modulators.

## Figures and Tables

**Table 1:** Parameters for the description according to Equations (1)–(3) of the Tris-T4-linked effect on the 20S chymotryptic-like catalytic activity.

	Human	Yeast	α3ΔN
k*_catA_* (s^−1^)	11.5 ± 1.3	0.95 ± 0.15	49.5 ± 5.6
k*_catB_* (s^−1^)	0.32 ± 0.05	0.17 ± 0.03	49.5 ± 5.6
K*_mA_* (M)	1.95(± 0.37) × 10^−3^	1.17(± 0.22) × 10^−4^	4.1(± 0.6) × 10^−3^
K*_mB_* (M)	1.5(± 0.3) × 10^−4^	9.04(± 1.82) × 10^−6^	1.07(± 0.27) × 10^−2^
K*_AU_*(M^−1^)	2.0(± 0.3) × 10^4^	3.0(± 0.5) × 10^5^	3.0(± 0.5) × 10^5^
K*_BU_*(M^−1^)	8.2(± 1.2) × 10^7^	9.0(± 1.8) × 10^7^	9.0(± 1.8) × 10^7^
K*_AL_*(M^−1^)	1.2(± 0.3) × 10^4^	1.2(± 0.3) × 10^4^	1.2(± 0.4) × 10^5^
K*_BL_* (M^−1^)	1.35(± 0.28) × 10^8^	1.3(± 0.3) × 10^8^	1.3(± 0.3) × 10^8^
K*_BI_* (M^−1^)	-	2.5(± 0.4) × 10^6^	2.5(± 0.4) × 10^6^
L*_U_*	3.0(± 0.5) × 10^−6^ (3)	6.0(± 1.0) × 10^−4^ (2)	3.9(± 0.6) × 10^−2^
L*_L_*	3.9(± 0.6) × 10^−5^ (3)	7.8(± 1.5) × 10^−3^ (2)	1.5(± 1.0) × 10^−2^

## Supplementary Materials

Supplementary Materials can be found at https://www.mdpi.com/1422-0067/21/19/7190/s1.

## Notes

^§^ In order to simulate the dynamics of the ligand-protein recognition event, we applied an original docking protocol based on a Monte Carlo-Metropolis search followed by a simulated annealing (SA) procedure [53,54,55]. Restraints were applied only to the backbone atoms of the calculated conserved secondary strucures (Table S15) (see the Experimental Section for details). A final unrestrained structure optimization was performed, and the generated complexes were checked for their structural quality by using the Molprobity structure evaluator software (Supplementary Table S3) [56]. Ligand-induced h20S conformational changes were analysed in detail for the selected complexes. The complexes with the best compromise between the nonbonded interaction energy obtained from Metropolis and SA calculations were selected as representatives of the most probable Tris-T4 binding modes (best-docked solutions) (Supplementary Tables S4–S7). The conformational energy of the ligand was evaluated and taken into account in the analysis of docking results (Supplementary Table S8).

## Abbreviations

UPS	ubiquitin-proteasome system
RPs	regulatory particles
CP	core particle
PCD	protein conformational disorders
ChT-L	chymotrypsin-like
T-L	trypsin-like
C-L	caspase-like
IDP	intrinsically disordered proteins
h20S	human 20S
y20S	yeast 20S
SARs	structure–activity relationships
STD	saturation transfer difference
WL	WaterLogsy
SA	simulated annealing
MM	molecular mechanics
RMSD	root mean square deviation
