# Supplementary material for: Cooperative Binding of the Cationic Porphyrin Tris-T4 Enhances Catalytic Activity of 20S Proteasome Unveiling a Complex Distribution of Functional States
Source: Int J Mol Sci. 2020 Sep 29;21(19):7190. doi: 10.3390/ijms21197190 (PMC7582714; doi:10.3390/ijms21197190)
Supplement: Supplementary file 1 [file ijms-21-07190-s001.pdf]

Supplementary Materials

Cooperative binding of the cationic porphyrin Tris-T4 enhances catalytic activity of 20S proteasome unveiling a complex distribution of functional states

Anna Maria Santoro [1], Alessandro D'Urso [2], Alessandra Cunsolo [2,3], Danilo Milardi [1], Roberto Purrello [2], Diego Sbardella [4], Grazia R. Tundo [5], Donatella Diana [6], Roberto Fattorusso [7], Antonio Di Dato [8], Antonella Paladino [9], Marco Persico [8,10], Massimo Coletta [5,*] and Caterina Fattorusso [8,10,*]

Table of Contents:

1. Experimental section: NMR spectroscopy and molecular modeling **S2-S6**
2. Molecular modelling studies (Tables S1-S15) **S7-S28**
3. NMR studies (Figures S1-S2) **S29-S30**
4. Molecular modelling studies (Figures S3-S14) **S31-S44**
5. Kinetic analyses (Figure S15) **S45-S46**
6. References **S47**

Experimental section.

NMR spectroscopy. All NMR spectra were recorded at 298K on a Varian Inova 600 MHz spectrometer, with a 5-mm inverse-detection cryioprobe equipped with z-gradient, located at IBB-CNR, Napoli.

The 1H NMR signal assignment of **Tris-T4** was obtained from the analysis of the spectra assisted by the theoretical prediction based in the molecular structure using ChemAxon software (http://www.chemaxon.com).

For interaction studies, samples (total volume, 250 µl) were prepared in 3 mm NMR tubes, using 0.860 mM of 20S and 175 mM of **Tris-T4** in 50 mM Hepes, 100 mM NaCl and 1mM DTT; ligand/20S molar ratio of 200:1 was used.

STD spectra were acquired with 512 scans with on-resonance irradiation at −0.5 ppm for selective saturation of protein resonances and off-resonance irradiation at 30 ppm for reference spectra. A train of 40 Gaussian-shaped pulses of 50 ms with 1 ms delay between pulses were used, for a total saturation time of 2 s. STD spectra were obtained by internal subtraction of the saturated spectrum from the reference spectrum by phase cycling with a spectral width of 7191.66 Hz, relaxation delay 1.0 s, 8k data points for acquisition, and 16k for transformation. WaterLOGSY NMR experiments employed a 20 ms selective Gaussian 180° pulse at the water signal frequency and a NOE mixing time of 1 s. Both for STD and WaterLOGSY, the FIDs were multiplied by an exponential weighting (lb = 5 Hz) before Fourier transformation.

1H and STD spectra of **Tris-T4** alone were acquired as reference.

1D 1H, STD and WaterLOGSY spectra were processed by means of the ChemAxon software (http://www.chemaxon.com)

The STD factor was calculated as $A_{STD} = (I_0 \;\; I_{sat})/I_0 = I_{STD}/I_0$ where I_0 is the intensity of the signal in the reference experiment and Isat is the intensity of the same signal in the saturated spectrum. The signal obtained with the strongest I_{STD}/I_0 value was normalized to 100%. The relative degree of saturation for the individual protons was used to compare the STD effect.

Calculation of the chemical-physical properties of Tris-T4. The apparent pKa values of **Tris-T4** were estimated by using the algorithm ACD/pKa GALAS (ACD/Percepta software, version 2017.1.3, Advanced Chemistry Development, Inc., Toronto, ON, Canada, 2017, http://www.acdlabs.com). The compound was considered in its cationic form in all calculations performed, as a consequence of the estimation of percentage of neutral/ionized forms computed at pH 7.2 (cytoplasmic value) using the Handerson−Hasselbalch equation. Atomic potentials were assigned using the CVFF force field, while the partial charges were assigned using the partial charges estimated by MNDO semi-empirical 1 SCF calculations[1]. The conformational space of the porphyrin was sampled through 200 cycles of simulated annealing (SA; ε =80*r) followed by molecular mechanics (MM) energy minimization. During the SA procedure, the temperature is altered in time increments from an initial temperature to a final temperature by adjusting the kinetic energy of the structure (by rescaling the velocities of the atoms). The following protocol was applied: the system was heated to 1000 K over 2000 fs (time step of 1.0 fs); a temperature of 1000 K was applied to the system for 2000 fs (time step of 1.0 fs) to surmount torsional barriers; successively, temperature was linearly reduced to 300 K in 1000 fs with a decrement of 0.5 K/fs (time step of 1.0 fs). Resulting conformations were then subjected to MM energy minimization within Insight 2005 Discover 3 module (CVFF force field; ε = 80*r) until the maximum rms was less than 0.001 kcal/Å, using conjugate gradient[2] as the minimization algorithm. The resulting MM conformers were subsequently ranked by: i) conformational energy (ΔE from the global energy minimum < 5 kcal/mol), ii) interatomic distances between the charged nitrogen atoms, and iii) conformation of porphyrin ring. In order to properly analyze the electronic properties, the conformers, obtained from molecular dynamics and mechanics calculations, were subjected to a full geometry optimization through semiempirical calculations, using the quantum mechanical method PM7[3] in the Mopac2012 package (Stewart Computational Chemistry: Colorado Springs, CO, USA; http://OpenMOPAC.net (2012).[4] The EF (Eigenvector Following routine) algorithm of geometry optimization was used[5], with a GNORM value set to 0.01. To reach a full geometry optimization, the criterion for terminating all optimizations was increased by a factor of 100, using the keyword PRECISE.

The resulting PM7 conformers were subsequently ranked as reported above for MM conformers. The global minimum conformer of **Tris-T4** was selected for the dynamic docking studies in complex with human 20S.

Docking studies on human 20S proteasome. Docking calculations were performed by using our previously developed atomic models of human 20S in the closed and open conformational states[5]. It has to be underlined that, since we used as template for human 20S in the open state the structure determined by Chen et al. by cryo-electron microscopy (cryoEM)[6], then, our atomic model of human 20S in the open state is composed by just one "half" (one ◎-ring and one ◎-ring) of the entire 20S structure.

The four starting structures to be used in docking studies were generated by positioning: i) one **Tris-T4** molecule at $\alpha5$-$\alpha6$ groove (closed state); ii) one **Tris-T4** at $\alpha4$-$\alpha5$ (open state); iii) three **Tris-T4** molecules bound at $\alpha1$-$\alpha2$, $\alpha4$-$\alpha5$ and $\alpha5$-$\alpha6$ grooves (closed and open states). The starting complexes were then subjected to dynamic docking studies (Affinity, SA_Docking; Insight2005, Accelrys, San Diego). In particular, a docking methodology, which considers all the systems flexible (i.e., ligand and protein), was used. Flexible docking was achieved using the Affinity module in the Insight 2005 suite, setting the SA_Docking procedure[7] and using the Cell Multipole method for non-bonded interactions[8].

The docking protocol included a Monte Carlo based conformational search of the ligand within the obtained homology models of human 20S proteasome (i.e., closed and open conformation) for the random generation of a maximum of 20 acceptable complexes. During the first step, in the starting structures, the ligand was moved by a random combination of translation, rotation, and torsional changes to sample both the conformational space of the ligand and its orientation with respect to the binding domain area (MxRChange = 3 Å; MxAngChange = 180°). The binding domain area was defined as a subset including all residues of human 20S proteasome. Thus, all proteasome atoms were left free to move during the entire course of docking calculations, whereas, in order to avoid unrealistic results during the subsequent SA calculations, a tethering restraint was applied on the SCRs of the protein (defined below).

During the Monte Carlo/Metropolis docking step, van der Waals (vdW) and Coulombic terms were scaled to a factor of 0.1 to avoid very severe divergences in the vdW and Coulombic energies. If the energy of a complex structure resulting from random moves of the ligand was higher by the energy tolerance parameter than the energy of the last accepted structure, it was not accepted for minimization. To ensure a wide variance of the input structures to be successively minimized, an energy tolerance value of 10^6 kcal/mol from the previous structure was used. After the energy minimization step (conjugate gradient; 2500 iterations; ε = 1), the energy test, with an energy range of 50 kcal/mol, and a structure similarity check (rms tolerance = 0.3 kcal/Å) was applied to select the 20 acceptable structures. Each subsequent structure was generated from the last accepted structure. Following this procedure, the resulting docked structures were ranked by their conformational energy and were analyzed considering the non-bonded interaction energies between the ligand and the enzyme (vdW and electrostatic energy contribution; Group Based method[9]; CUT_OFF = 100; ε = 2*r; Discover_3 Module of Insight2005).

The Monte Carlo docked complexes were then subjected to molecular dynamics simulations at flexible temperatures (Simulated Annealing, SA) to enhance the fixing of the ligand into the binding site and to explore possible ligand-induced large-scale conformational changes of the protein. In particular, the resulting docked complexes were subjected also to a molecular dynamics SA protocol using the Cell_Multipole method for non-bonded interactions and the dielectric constant of the water (ε = 80*r). A tethering restraint was applied on the SCRs of the complex. The set of structural restraints applied was the same as for previous docking calculations. The protocol included 5 ps of a dynamic run divided in 50 stages (100 fs each) during which the temperature of the system was linearly decreased from 500 to 300 K (Verlet velocity integrator; time step = 1.0 fs). In simulated annealing, the temperature is altered in time increments from an initial temperature to a final temperature. The temperature is changed by adjusting the kinetic energy of the structure (by rescaling the velocities of the atoms). Molecular dynamics calculations were performed using a constant temperature and constant volume (NVT) statistical ensemble, and the direct velocity scaling as temperature control method (temp

window = 10 K). In the first stage, initial velocities were randomly generated from the Boltzmann distribution, according to the desired temperature, while during the subsequent stages initial velocities were generated from dynamics restart data. The temperature of 500 K was applied with the aim of surmounting torsional barriers, thus allowing an unconstrained rearrangement of the "ligand" and the "protein" binding site (initial vdW and Coulombic scale factors = 0.1). Successively temperature was linearly reduced to 300 K in 5 ps, and, concurrently, the vdW and Coulombic scale factors have been similarly increased from their initial values (0.1) to their final values (1.0). A final round of 10^5 minimization steps ($\varepsilon = 80*r$) followed the last dynamics steps, and the minimized structures were saved in a trajectory file. The ligand/enzyme complexes thus obtained were ranked by their conformational energy and analyzed considering the non-bonded interaction energies between the ligand and the enzyme (vdW and electrostatic energy contribution; Group Based method; CUT_OFF = 100; $\varepsilon = 2*r$; Discover_3 Module of Insight2005). The complex with the best compromise between the non-bonded interaction energies obtained by Monte Carlo and SA calculations was selected as the structure representing the most probable binding mode. In order to allow the whole relaxation of the protein, the selected annealed complexes were then subjected to MM energy minimization without restraints (Steepest Descent algorithm; $\varepsilon = 80*r$) until the maximum RMS derivative was less than 0.5 kcal/Å (Module Discover; Insight 2005). The protein structural quality in the resulting complexes was then checked using Molprobity structure evaluator software[10] and compared to that of the reference PDB structure.

The SCRs of the human 20S proteasome were identified using the Structure Prediction and Sequence Analysis server PredictProtein (http://www.predictprotein. org/) and are reported in Table S15. Within the identified SCRs, the following restraints were used: the distance between backbone hydrogen bond donors and acceptors in the alpha-helices was restrained within 2.5 Å. On the other hand, the φ and ψ torsional angles of the beta-sheets were restrained to -119° and +113°, or -139° and +135°, respectively, according to the presence of a parallel or anti-parallel structure. In particular, according to the reliability index values obtained from the secondary structure prediction, the following set

of force constant values were applied (quadratic form) : i) 1 kcal/mol/Å^2 (maximum force: 10 kcal/mol/Å^2) for reliability index values from 0 to 3, ii) 10 kcal/mol/Å^2 (maximum force: 100 kcal/mol/Å^2) for reliability index values from 4 to 6, and iii) 100 kcal/mol/Å^2 (maximum force: 1000 kcal/mol/Å^2) for reliability index values from 7 to 9.

Structural investigation of docked complexes. In order to evaluate proteasome structure atomic position variations upon TrisT4 binding Root Mean Square Deviations (RMSD) were calculated on Cα atoms by comparing each structure to a reference structure: both the closed and the open conformation were used as reference structure. To this end proteasome structures from all docking calculations including starting conformations were collected and analyzed (39 frames; see Tables 3-6SI). RMSDs were computed taking into account both α and β rings and performed using GROMOS (GROningen MOlecular Simulation computer program package; www.gromos.net).

Structural analyses of the docked complexes were performed using Macromolecules and Receptor-Ligand Interaction tools of Discovery Studio 2017 (Dassault Systèmes BIOVIA, San Diego, 2017). Sequence alignments of *yeast* and human 20S proteasome subunits were performed using PROMALS3D server (http://prodata.swmed.edu/promals3d/promals3d.php).[11] In order to identified the negatively charged ionic residues of h20S involved in the interaction with the RPs and the hydrophobic residues of h20S involved in the interaction with the C-terminal tails of RPs, the previous structural analysis[5] was implemented using the same procedure on the new experimentally determined structures of h20S proteasome in complex with the regulatory particle 19S (PDB ID: 6MSB, 6MSD, 6MSE, 6MSG, 6MSH, 6MSJ and 6MSK) and PA200 (PDB ID: 6WKY and 6REY). These structures were downloaded from the Protein Data Bank (PDB; http://www.rcsb.org/pdb/) and were analyzed using Biopolymer and Homology modules of Insight 2005 (Accelrys, San Diego).

CAVER[12] was used to identify tunnels considered as void pathways leading from a cavity buried in protein core to the bulk solvent. Tunnels were generated selecting as starting point the center of gravity of all residues within 5 Å from the β5-Thr1 (i.e, R19, A20, V31, K33,

A46, G47, G48, A49, C52) (max distance = 3.0 Å and desired radius = 5.0 Å). Tunnels were generated using the following parameters: 0.9 Å for probe radius (minimum radius of the tunnel), 2.0 Å for shell depth (maximal depth of a surface region) and 3.0 Å for shell radius (radius of the shell probe used to define the bulk solvent). Tunnel clustering was performed by the average-link hierarchical algorithm selecting 2.0 Å for the calculation of pairwise tunnel distances (i.e. dissimilarities) as the cutoff able to capture all the representative directions of the identifies tunnels.

Solvent accessible surface area (SASA) calculations were performed using Discovery Studio 2017 (Dassault Systèmes BIOVIA, San Diego, 2017). The SASA of each Tris-T4 molecule bound to h20S in the selected docked complexes (starting either from the open or the closed protein conformation) was calculated and compared to the SASA of the unbound molecule. The rate of SASA decrease was calculated by using Microsoft Excel.

Table S1. Calculated pka values and ionic forms of **Tris-T4** (ACD/pKa GALAS; ACD/Percepta software).

Compound	pka1	pka2	Ionic forms at pH 7.2 (%)
**Tris-T4**	6.6±1.1	11.3±1.2	Tetra-cationic (21)<br>Tri-cationic (79)

Table S2. Identified clusters of three negatively charged amino acids present on the α-ring surface of human 20S proteasome in closed and open conformation able to interact with **Tris-T4** and related inter-residue distances (Cα).

	Closed state		Open State	
**Groove**	**d1 (Å)**	**d2(Å)**	**d1 (Å)**	**D2(Å)**
**α1-α2**	14.07<br>E19(α1)-E26(α1)	10.41<br>E26(α1)-D155(α1)	13.23<br>E19(α1)-E26(α1)	11.72<br>E26(α1)-D155(α1)
**α4-α5 (I)**	18.64<br>D13(α4)-E24(α4)	6.24<br>D13(α4)-E29(α5)	19.47<br>D13(α4)-E24(α4)	7.16<br>D13(α4)-E29(α5)
**α4-α5 (II)**	13.21<br>E20α4)-D13(α4)	6.24<br>D13(α4)-E29(α5)	12.74<br>E20α4)-D13(α4)	7.16<br>D13(α4)-E29(α5)
**α5-α6 (I)**	19.54<br>E29(α5)-E18(α5)	6.10<br>E18(α5)-E27(α6)	-	-
**α5-α6 (II)**	11.48<br>E25(α5)-E18(α5)	6.10<br>E18(α5)-E27(α6)	14.52<br>E25(α5)-E18(α5)	7.86<br>E18(α5)-E27(α6)

Table S3. Summary of Molprobity results obtained for the experimentally determined structures of human 20S proteasome, their full-length atomic models and the docked porphyrin/20S complexes.

Structure	Residues favored regions	Residues allowed regions	Residues outliers	Poor rotamers
X-ray (4R3O) (Closed State)	94.9%	4.3%	0.8%	4.3%
Cryo-EM (5T0J) (Open State)	92.1%	7.7 %	0.2%	0.9%
Molecular model (Closed State)	94.8%	4.3%	0.9%	4.1%
Molecular model (Open State)	91.4%	8.2%	0.4%	1.3%
**Tris-T4** Cplx_1 (3 molecules; starting: closed state)	78.9%	18.1%	3.0%	2.1%
**Tris-T4** Cplx_1 (3 molecules; starting: open state)	80.3%	16.6%	3.1%	2.1%
**Tris-T4** Cplx_5 (1 molecule; starting: closed state)	79.7%	17.4%	2.9%	2.1%
**Tris-T4** Cplx_4 (1 molecule; starting: open state)	77.8%	18.8%	3.4%	1.7%

Table S4. Nonbonded interaction energies (kcal/mol) of the 20S-**Tris-T4** complexes obtained by Monte Carlo and SA calculations using as starting binding site the α5-α6 groove of 20S in the closed conformation.

**Cplx**	**Nonbonded interaction energies (kcal/mol)**					
	Monte Carlo Simulation			Simulated Annealing Simulation		
	vdW	Coulomb	Total	vdW	Coulomb	Total
**1**	-25.469	-13.735	-39.204	-41.669	-7.209	-48.877
**2**	-27.596	-13.286	-40.883	-36.189	-7.482	-43.672
**3**	-22.867	-14.792	-37.659	-36.410	-16.741	-53.151
**4**	-17.667	-18.392	-36.059	-35.085	-15.979	-51.064
**5**[a]	-23.333	-14.690	-38.023	-51.398	-4.093	-55.491
**6**	-0.527	-2.558	-3.085	-33.174	0.962	-32.212
**7**	-8.953	-7.614	-16.567	-51.291	-6.460	-57.751
**8**	-24.361	-14.594	-38.955	-47.097	-2.378	-49.475
**9**	-33.095	-6.576	-39.671	-56.979	-7.744	-64.723
**10**	-25.005	-10.483	-35.487	-43.958	-0.951	-44.909
**11**	-0.525	-2.739	-3.264	-43.364	9.964	-33.399

[a]Selected complexes

Table S5. Nonbonded interaction energies (kcal/mol) of the 20S-**Tris-T4** complexes obtained by Monte Carlo and SA calculations using as starting binding site the α4-α5 groove of 20S in the open conformation.

Cplx	Nonbonded interaction energies (kcal/mol)					
	Monte Carlo Simulation			Simulated Annealing Simulation		
	vdW	Coulomb	Total	vdW	Coulomb	Total
**1**	-18.783	-8.860	-27.643	-53.566	2.540	-51.025
**2**	-18.005	-8.961	-26.965	-57.454	-0.996	-58.450
**3**	-16.783	-11.143	-27.928	-42.356	-9.547	-51.903
**4**[a]	-16.636	-10.984	-27.620	-51.230	-9.600	-60.831
**5**	-3.187	-7.140	-10.327	-62.339	-6.105	-68.444
**6**	2.679	-7.993	-5.315	-27.843	-4.341	-32.184
**7**	-5.179	-3.223	-8.401	-56.803	-2.897	-59.700
**8**	-5.508	-5.007	-10.514	-46.283	-4.819	-51.103
**9**	-2.834	-3.978	-6.812	-41.699	1.654	-40.045

[a]Selected complex

Table S6. Nonbonded interaction energies (kcal/mol) of the 20S in complex with three molecules of **Tris-T4** obtained by Monte Carlo and SA calculations using as starting binding sites the α1-α2, α4-α5 and α5-α6 grooves of 20S in the closed conformation.

**Cplx**	**Nonbonded interaction energies (kcal/mol)**					
	Monte Carlo Simulation			Simulated Annealing Simulation		
	vdW	Coulomb	Total	vdW	Coulomb	Total
**1**[a]	-98.800	-41.517	-140.317	-171.108	5.795	-165.313
**2**	-22.935	-18.057	-40.992	-160.303	3.802	-156.501
**3**	-78.377	-35.529	-113.907	-157.977	10.189	-147.788
**4**	-60.500	-34.477	-94.977	-151.422	-1.875	-153.297
**5**	-55.782	-40.735	-96.517	-83.516	-15.652	-99.168
**6**	-77.702	-15.237	-92.938	-120.808	13.206	-106.874
**7**	-78.997	-17.388	-96.385	-143.897	12.652	-131.245
**8**	-81.693	-28.753	-110.446	-155.235	-7.881	-163.116
**9**	-66.203	-17.685	-83.888	-161.086	4.435	-156.651

[a]Selected complex

Table S7. Nonbonded interaction energies (kcal/mol) of the 20S in complex with three molecules of **Tris-T4** obtained by Monte Carlo and SA calculations using as starting binding sites the α1-α2, α4-α5 and α5-α6 grooves of 20S in the open conformation.

**Cplx**	**Nonbonded interaction energies (kcal/mol)**					
	Monte Carlo Simulation			Simulated Annealing Simulation		
	vdW	Coulomb	Total	vdW	Coulomb	Total
**1**[a]	-66.387	-18.887	-85.274	-144.197	3.380	-140.817
**2**	-4.039	7.431	3.392	-116.600	-5.287	-121.887
**3**	-5.680	-0.063	-5.743	-143.156	7.652	-135.504
**4**	-9.199	-3.893	-13.093	-131.408	22.359	-109.049
**5**	-1.246	-5.385	-6.631	-40.043	-11.613	-51.656
**6**	-3.016	4.501	1.484	-89.168	12.507	-76.661
**7**	-15.019	-3.025	-18.044	-133.642	-10.110	-143.752
**8**	-2.448	3.231	0.783	-145.664	-12.591	-158.255

[a]Selected complex

Table S8. Conformational energies (◎E from the Global Minimum Conformer) of **Tris-T4** docked on human 20S proteasome.

**Complex**	**ΔE_{GM} (kcal/mol)**		
	**TrisT4_1**	**TrisT4_2**	**TrisT4_3**
20S Closed /1 TrisT4	5.54 (α5-α6)	-	-
20S Open /1 TrisT4	-	3.70 (α4-α5)	-
20S Closed /3 TrisT4	4.10 (α5-α6)	3.12 (α4-α5)	9.48 (α1-α2)
20S Open /3 TrisT4	7.73 (α5-α6)	8.35 (α4-α5)	10.57 (α1-α2)

Table S9. Ligand-residue non-bond interaction energies (kcal/mol) of the **Tris-T4**/h20S complex obtained by Monte Carlo and SA calculations using as starting binding site the α5-α6 groove of 20S in the closed conformation. The residues involved in the interaction with RPs are noted and the RPs are reported. The corresponding *wt y*20S residues (PROMALS3D alignment; all conserved in the α3ΔN mutant) are also listed.

*h*20S amino acids	*y*20S amino acids[a]	Subunit	Non-bond interaction Energy (kcal/mol)	RPs[b]
R20[c]	R20	α5	-0.545	19S (EA1-2, EB, EC1-C2, ED1-2) and PA200
V24[c]	V24	α5	-1.110	19S (EA1-2, EB, EC1-2 and ED1-2) and PA200
E25[c,d]	E25	α5	-2.564	19S (EA1-2, EB, SC/EC1-2 and SD/ED1-2), PA28 and PA200
**I28**[c]	**L28**	**α5**	-2.889	19S (EC1-2, ED1-2) and PA200
E29[d]	E29	α5	-7.587	19S (SA/EA1-2)
A30	A30	α5	-0.500	
**D157**[c]	**E158**	**α5**	-1.179	19S (EB) and PA200
A169	A170	α5	-1.766	
I170	I171	α5	-0.717	
G171	G172	α5	-0.138	
S174	S175	α5	-1.259	
E175	E176	α5	-10.131	
G176	G177	α5	-0.637	
Q178	Q179	α5	-3.957	
**S179**	**A180**	**α5**	-3.222	
**Q182**	**L183**	**α5**	-3.400	
**E183**	**N184**	**α5**	-6.065	
**Q53**[c]	**A53**	**α6**	-2.086	19S (EA1-2, EB, EC1-2, ED1-2) and PA200
**S54**	**D54**	**α6**	-2.117	
E55	E55	α6	-3.353	
L56	L56	α6	-0.399	
**Q203**	**S205**	**α6**	-1.083	

[a] Both the wild-type and the α3ΔN mutant; the amino acids not conserved between human and yeast 20S proteasome (alignment performed using PROMALS3D server) are evidenced in bold. [b] 19S functional states involved in the reported interaction are specified in brackets. [c]Amino acids of the C-terminal tail of Rpt5 (aa426-aa439; α5/α6) (19S) and PA200 (aa1830-aa1843) having at least one atom within a 4 Å radius from any given h20S residue. [d] Negatively charged residues involved in ionic interaction with RPs (i.e., PA28, PA200 and 19S).

Table S10. Ligand-residue non-bond interaction energies (kcal/mol) of the **TrisT4**-20S complex obtained by Monte Carlo and SA calculations using as starting binding site the α4-α5 groove of 20S in the open conformation; The residues involved in the interaction with RPs are noted and the RPs are reported. The corresponding *wt y*20S residues (PROMALS3D alignment; all conserved in the α3ΔN mutant) are also listed.

*h*20S amino acids	*y*20S amino acids[a]	Subunit	Non-bond interaction Energy (kcal/mol)	RPs[b]
D13[c,d]	D14	α4	-6.607	19S (E_{D1}) and PA28
G14[c]	G15	α4	-0.917	19S (E_{D1-2})
H15[c]	H16	α4	-0.481	19S (E_{D1-2})
E20[c,d]	E21	α4	-7.593	19S (E_{D1-2}) and PA28
**Q23**[c]	**L24**	**α4**	-2.349	19S (E_{D1-2})
E24[c,d]	E25	α4	-7.463	19S (S_A/E_{A1-2} S_B/E_B, E_{D1-2})
T147	T150	α4	-0.164	
**D148**[c]	**E151**	**α4**	-5.178	19S (E_{D2})
P149	P152	α4	-0.458	
S150[c]	S153	α4	-0.878	19S (E_{D1-2})
G151	G154	α4	-0.486	
**T152**[c]	**I155**	**α4**	-2.562	19S (E_{D1-2})
G162	G165	α4	-0.241	
**G164**	**N167**	**α4**	-0.451	
E29[d]	E29	α5	-2.363	19S (S_A/E_{A1-2})
I31	I31	α5	-0.715	
K32	K32	α5	-2.271	
L33[c]	L33	α5	-5.769	19S (E_{D1-2})
G34[c]	G34	α5	-2.942	19S (E_{D1-2})
S35[c]	S35	α5	-0.929	19S (E_{D1-2})
T36	T36	α5	-0.630	
E51[d]	E51	α5	-4.263	19S (S_A, S_B)
R53[c]	R53	α5	-0.153	19S (E_{D1-2})
G80[c]	G80	α5	-0.148	19S (E_{D1-2})
L81[c]	L81	α5	-0.088	19S (E_{D1-2})
E207[d]	E209	α5	-5.963	19S (S_A/E_{A1-2}; S_B/E_B and S_C/E_{C1-2})

[a] Both the wild-type and the α3ΔN mutant; the amino acids not conserved between human and yeast 20S proteasome (alignment performed using PROMALS3D server) are evidenced in bold. [b] 19S functional states involved in the reported interaction are specified in brackets. [c]Amino acids of the C-terminal tail of Rpt1 (aa421-aa433; α4/α5) (19S) having at least one atom within a 4 Å radius from any given h20S residue. [d]Negatively charged residues involved in ionic interaction with RPs (i.e., PA28, PA200 and 19S).

Table S11. Ligand-residue non-bond interaction energies (kcal/mol) of the 20S in complex with three molecules of **Tris-T4** obtained by Monte Carlo and SA calculations using as starting binding sites the α5-α6, α4-α5 and α1-α2 grooves of 20S in the closed conformation. The residues involved in the interaction with RPs are noted and the RPs are reported. The corresponding *wt y*20S residues (PROMALS3D alignment; all conserved in the α3ΔN mutant) are also listed.

Molecule	*h*20S amino acids	*y*20S amino acids[a]	Subunit	Non-bond interaction Energy (kcal/mol)	RPs[b]
**TrisT4_1 (α5/α6)**	F15[c]	F15	α5	-0.160	19S (E_{C2})
	E18[c,d]	E18	α5	-1.182	19S (S_A/E_{A1-2}; S_B/E_B; S_C; S_D/E_{D1-2}), PA28 and PA200
	G19[c]	G19	α5	-0.556	19S (E_{C1-2}, E_{D1-2}) and PA200
	L21[c]	L21	α5	-0.393	19S (E_{A1-2}, E_B, E_{C1-2}, E_{D1-2}) and PA200
	E25[c, d]	E25	α5	-2.568	19S (E_{A1-2}, E_B, S_C/E_{C1-2} and S_D/E_{D1-2}), PA28 and PA200
	E29[d]	E29	α5	-4.414	19S (S_A/E_{A1-2})
	**D157**[c]	**E158**	α5	-6.356	19S (E_B) and PA200
	P158	P159	α5	-0.107	
	S159[c]	S160	α5	-1.347	19S (E_{A1-2}, E_B, E_{C1-2}, E_{D1-2}) and PA200
	G160	G161	α5	-0.316	
	T161[c]	T162	α5	-1.688	19S (E_{A1-2}, E_B, E_{C1-2}, E_{D1}) and PA200
	F162	F163	α5	-0.545	
	**V163**	**Y164**	α5	-1.806	
	A28[c]	A28	α6	-0.989	19S (E_{A1-2}, E_B, E_{C1-2} and E_{D1-2}) and PA200
	Q31[c]	Q31	α6	-3.634	19S (E_{A1-2}, E_B, E_{C1-2}, E_{D1-2}) and PA200
	G32[c]	G32	α6	-2.111	19S (E_{A1-2}, E_B, E_{C1-2} and E_{D1-2}) and PA200
	S33[c]	S33	α6	-0.392	19S (E_{A1-2}, E_B, E_{C1-2}, E_{D1-2}) and PA200
	**A52**[c]	**N52**	α6	-0.448	19S (E_{C1}, E_{D1-2})
	Q53[c]	A53	α6	-2.084	19S (E_{A1-2}, E_B, E_{C1-2}, E_{D1-2}) and PA200
	G76[c]	G76	α6	-0.716	19S (E_{A1-2}, E_B, E_{C1-2}, E_{D1-2}) and PA200
	L77[c]	L77	α6	-1.810	19S (E_{A1-2}, E_B, E_{C1-2}, E_{D1-2}) and PA200
	**T78**[c]	**A78**	α6	-0.488	19S (E_{A1-2}, E_B, E_{C1-2}, E_{D1-2}) and PA200
	A163[c]	A63	α6	-1.824	19S (E_{C2}) and PA200
	**E197**	**Q199**	α6	-1.237	
	**T198**	**S200**	α6	-1.416	
	L199	L201	α6	-1.518	
	**P200**	**R202**	α6	-0.602	

	**Q203**	**S205**	α6	-2.517	
	D204[d]	-	α6	-8.103	PA200
	L205	L206	α6	-0.448	
**TrisT4_2 (α4/α5)**	Q146	Q149	α4	-0.377	
	**D148**	**E151**	α4	-2.164	
	**T152**[c]	**I155**	α4	-0.524	19S (E_{D1-2})
	**H154**	**S157**	α4	-2.177	
	W156[c]	W159	α4	-1.409	19S (E_{D1-2})
	A158	A161	α4	-0.497	
	**N159**	**Q162**	α4	-1.120	
	**A160**	**T163**	α4	-0.754	
	**G164**	**N167**	α4	-0.641	
	**A165**[c]	**S168**	α4	-1.294	19S (E_{D2})
	E170[d]	E173	α4	-3.668	PA200
	E173	E176	α4	-3.453	
	A30	A30	α5	-0.322	
	I31	I31	α5	-0.764	
	L33[c]	L33	α5	-4.628	19S (E_{D1-2})
	G34[c]	G34	α5	-1.624	19S (E_{D1-2})
	S35[c]	S35	α5	-0.416	19S (E_{D1-2})
	R53[c]	R53	α5	-0.605	19S (E_{D1-2})
	T55[c]	T55	α5	-0.468	19S (E_{D1-2})
	G80[c]	G80	α5	-0.421	19S (E_{D1-2})
	L81[c]	L81	α5	-0.754	19S (E_{D1-2})
	**I82**[c]	**T82**	α5	-1.766	19S (E_{D1-2})
	G171	G173	α5	-0.175	
	S172	S174	α5	-2.659	
	**A173**	**G175**	α5	-1.043	
	S174	S176	α5	-0.127	
	Q204	Q206	α5	-3.181	
	V205	V207	α5	-3.203	
	M206	M208	α5	-2.220	
	E207[d]	E209	α5	-5.295	19S (S_A/E_{A1-2}; S_B/E_B and S_C/E_{C1-2})
	E208	E210	α5	-2.889	
**TrisT4_3 (α1/α2)**	G20	G23	α1	-0.647	
	L22[c]	L25	α1	-1.152	19S (E_{A1-2}, E_B, E_{C1-2}, E_{D1-2})
	V25[c]	V28	α1	-1.116	19S (E_{A1-2}, E_B, E_{C1-2}, E_{D1-2})
	E26[c, d]	E29	α1	-7.734	19S (E_{A1-2}, E_B, E_{C1-2}, E_{D1-2}) and PA28
	F29[c]	F32	α1	-8.420	19S (E_{A1-2}; E_B, E_{C1-2}, E_{D1-2})
	**I32**	**T35**	α1	-0.205	
	N33	N36	α1	-2.428	
	Q34	Q37	α1	-2.107	
	S39	S42	α1	-0.368	
	**V40**	**L43**	α1	-0.084	
	D155[c]	D158	α1	-5.244	19S (E_{A1-2})
	G158	G161	α1	-0.189	
	Y159[c]	Y162	α1	-7.923	19S (E_{A1-2}, E_B, E_{C1-2}, E_{D1-2})
	Y160	Y163	α1	-2.433	
	**C161**[c]	**V164**	α1	-1.978	19S (E_{C1})
	G162	G165	α1	-0.192	
	**F163**	**Y166**	α1	-3.024	

T166	T169	α1	-0.532	
A167	A170	α1	-0.207	
**A168**	**T171**	α1	-2.739	
G169	G172	α1	-2.613	
**V170**	**P173**	α1	-0.773	
Q172	Q175	α1	-4.336	
A27	T27	α2	-0.315	
A28[c]	A28	α2	-0.306	19S (E_{C1}, E_{D2})
**A30**	**K30**	α2	-0.162	
**G31**[c]	**Q31**	α2	-0.895	19S (E_{A1-2}, E_B, E_{C2}, E_{D1-2})
G32[c]	G32	α2	-2.629	19S (E_{A1-2}, E_B, E_{C1-2}, E_{D1-2})
**A33**[c]	**V33**	α2	-0.647	19S (E_{A1-2}, E_B, E_{C1-2} and E_{D1-2})
K51[c]	K51	α2	-0.759	19S (E_{C1-2}, E_{D2})
**Q52**[c]	**S52**	α2	-1.187	19S (E_{D1})
E59	E60	α2	-1.987	
**S61**	**L62**	α2	-0.644	
**V62**	**S63**	α2	-0.135	
G78[c]	G79	α2	-0.212	19S (E_{A1-2}, E_B, E_{C1-2}, E_{D1-2})
G201	G204	α2	-0.256	
**Q202**	**E205**	α2	-4.364	

[a] Both the wild-type and the α3ΔN mutant; the amino acids not conserved between human and yeast 20S proteasome (alignment performed using PROMALS3D server) are evidenced in bold. [b] 19S functional states involved in the reported interaction are specified in brackets. [c] Amino acids of the C-terminal tail of Rpt5 (aa426-aa439; α5/α6), Rpt1 (aa421-433; α4/α5), Rpt3 (aa407-418; α1/α2) (19S) and PA200 (aa1830-aa1843) having at least one atom within a 4 Å radius from any given h20S residue. [d] Negatively charged residues involved in ionic interaction with RPs (i.e., PA28, PA200 and 19S).

Table S12. Ligand-residue non-bond interaction energies (kcal/mol) of the 20S in complex with three molecules of **Tris-T4** obtained by Monte Carlo and SA calculations using as starting binding sites the α5-α6, α4-α5 and α1-α2 grooves of 20S in the open conformation. The residues involved in the interaction with RPs are noted and the RPs are reported. The corresponding *wt y*20S residues (PROMALS3D alignment; all conserved in the α3ΔN mutant) are also listed.

Molecule	*h*20S amino acids	*y*20S amino acids[a]	Subunit	Non-bond interaction Energy (kcal/mol)	RPs[b]
**TrisT4_1 (α5/α6)**	F15[c]	F15	α5	-0.737	19S (E_{C2})
	P17	P17	α5	-0.669	
	E18[c,d]	E18	α5	-1.428	19S (S_A/E_{A1-2}; S_B/E_B; S_C; S_D/E_{D1-2}), PA28 and PA200
	G19[c]	G19	α5	-1.401	19S (E_{C1-2}, E_{D1-2}) and PA200
	R20[c]	R20	α5	-0.060	19S (E_{A1-2}, E_B and E_{C1-2}, E_{D1-2}) and PA200
	L21[c]	L21	α5	-2.384	19S (E_{A1-2}, E_B, E_{C1-2}, E_{D1-2}) and PA200
	V24[c]	V24	α5	-3.995	19S (E_{A1-2}, E_B, E_{C1-2} and E_{D1-2}) and PA200
	E25[c,d]	E25	α5	-5.458	19S (E_{A1-2}, E_B, S_C/E_{C1-2} and S_D/E_{D1-2}), PA28 and PA200
	Y26	Y26	α5	-0.542	
	**I28**[c]	**L28**	α5	-1.409	19S (E_{C1-2}, E_{D1-2}) and PA200
	E29[d]	E29	α5	-5.811	19S (S_A/E_{A1-2})
	**D157**[c]	**E159**	α5	-2.870	19S (E_B) and PA200
	S159[c]	S161	α5	-0.028	19S (E_{A1-2}, E_B, E_{C1-2}, E_{D1-2}) and PA200
	A169	A171	α5	-0.344	
	I170	I172	α5	-0.170	
	A28[c]	A28	α6	-1.582	19S (E_{A1-2}, E_B, E_{C1-2} and E_{D1-2}) and PA200
	**V29**	**I29**	α6	-0.526	
	Q31[c]	Q31	α6	-3.956	19S (E_{A1-2}, E_B, E_{C1-2}, E_{D1-2}) and PA200
	G32[c]	G32	α6	-2.158	19S (E_{A1-2}, E_B, E_{C1-2} and E_{D1-2}) and PA200
	S33[c]	S33	α6	-0.340	19S (E_{A1-2}, E_B, E_{C1-2}, E_{D1-2}) and PA200
	**A34**[c]	**V34**	α6	-0.107	PA200
	**A52**[c]	**N52**	α6	-3.273	19S (E_{C1}, E_{D1-2})
	**Q53**[c]	**A53**	α6	-7.749	19S (E_{A1-2}, E_B, E_{C1-2}, E_{D1-2}) and PA200
	**S54**	**D54**	α6	-1.288	
	E55	E55	α6	-7.095	
	L56	L56	α6	-1.559	
	**A57**	**S57**	α6	-0.286	
	G76[c]	G76	α6	-0.323	19S (E_{A1-2}, E_B, E_{C1-2}, E_{D1-2}) and PA200

	L77[c]	L77	α6	-0.725	19S (E_{A1-2}, E_B, E_{C1-2}, E_{D1-2}) and PA200
	I161	I161	α6	-0.419	
	L205	L206	α6	-0.367	
**TrisT4_2 (α4/α5)**	D13[c,d]	D14	α4	-6.862	19S (E_{D1}) and PA28
	G14[c]	G15	α4	-1.351	19S (E_{D1-2})
	H15[c]	H16	α4	-4.261	19S (E_{D1-2})
	**L16[c]**	**I17**	α4	-0.813	19S (E_{D2})
	F17	F18	α4	-2.093	
	V19	V20	α4	-0.309	
	E20[c,d]	E21	α4	-11.329	19S (E_{D1-2}) and PA28
	Y21	Y22	α4	-5.754	
	Q23[c]	L24	α4	-2.031	19S (E_{D1-2})
	E24[c,d]	E25	α4	-9.105	19S (S_A/E_{A1-2}, S_B/E_B, E_{D1-2})
	Q146[c]	Q149	α4	-0.825	19S (E_{D1-2})
	**H154**	**S157**	α4	-2.214	
	W156[c]	W159	α4	-1.824	19S (E_{D1-2})
	**A160**	**T163**	α4	-0.267	
	S35[c]	S35	α5	-1.202	19S (E_{D1-2})
	T36	T36	α5	-0.817	
	V50	V50	α5	-0.228	
	E51	E51	α5	-2.093	
	S79[c]	S79	α5	-0.860	19S (E_{D1})
	G80[c]	G80	α5	-0.144	19S (E_{D1-2})
	**I82[c]**	**T82**	α5	-0.079	19S (E_{D1-2})
**TrisT4_3 (α1/α2)**	**C161[c]**	**V164**	α1	-0.123	19S (E_{C1})
	**F163**	**Y166**	α1	-1.500	
	A165	A168	α1	-2.026	
	T166	T169	α1	-1.676	
	A167	A170	α1	-2.756	
	**A168**	**T171**	α1	-0.628	
	**V170**	**P173**	α1	-1.987	
	Q172	Q175	α1	-1.660	
	**T173[c]**	**Q176**	**α1**	-2.202	19S (E_{A1-2}, E_{C1-2})
	T176	T179	α1	-1.643	
	E180	E183	α1	-3.787	
	**Q52**	**S52**	α2	-2.422	
	S54	S54	α2	-2.747	
	L56	L56	α2	-0.999	
	**Y57[c]**	**M58**	α2	-2.438	19S (E_B, E_{C1-2}, E_{D1-2})
	**D58**	**S59**	α2	-0.788	

[a] Both the wild-type and the α3ΔN mutant; the amino acids not conserved between human and yeast 20S proteasome (alignment performed using PROMALS3D server) are evidenced in bold. [b] 19S functional states involved in the reported interaction are specified in brackets. [c]Amino acids of the C-terminal tail of Rpt5 (aa426-aa439; α5/α6), Rpt1 (aa421-433; α4/α5), of Rpt3 (aa407-418; α1/α2) (19S) and PA200 (aa1830-aa1843) having at least one atom within a 4 Å radius from any given h20S residue. [d] Negatively charged residues involved in ionic interaction with RPs (i.e., PA28, PA200 and 19S).

Table S13. Calculated rate of solvent accessible surface area (SASA) decrease for the phenyl and N-methyl-pyridyl hydrogen atoms of **Tris-T4** bound to h20S in the selected docking solutions.

	**SASA decrease (%)**					
	Starting h20S conformation: closed			Starting h20S conformation: open		
	α1-α2	α4-α5	α5-α6	α1-α2	α4-α5	α5-α6
para-Phe	100	0	100	100	100	100
meta-Phe	100	48	100	100	81	95
o*rtho*-Phe	75	47	43	50	43	61
meta N-Me-Pyr	77	67	42	20	40	64
ortho N-Me-Pyr	80	50	47	31	47	47

Table S14. Salt bridges and charge-assisted hydrogen bonds involving the N-terminals, α5-loop, and α6-loop present in the experimentally determined structures of of yeast 20S proteasome, wt closed (PDB ID: 1RYP), wt open (PDB ID: 1Z7Q) and the α3ΔN mutant (PDB ID: 1G0U).

Structure	Interactions
yeast **20S** *closed*	E125α5-R4α3
	E125α5-Y123α6
	S128 α5-Y123 α6
	R122 α5-E130α5
	R136α5-D84α5
yeast **20S** *open*	R132 α5-E130α5
	R136α5-D84α5
	S128 α5-Y123 α6
***α3ΔN mutant* 20S**	R130α5-D84α5

Table S15. Identified structurally conserved regions (SCRs) of the human 20S proteasome using the Structure Prediction and Sequence Analysis server PredictProtein (http://www.predictprotein. org/).

Subunits	Amino acid numbering	Secondary structure
α1	13-16	β-sheet
	23-33	α-helix
	38-43	β-sheet
	48-53	β-sheet
	68-72	β-sheet
	76-80	β-sheet
	85-104	α-helix
	111-128	α-helix
	135-143	β-sheet
	150-154	β-sheet
	160-168	β-sheet
	172-183	α-helix
	191-207	α-helix
	215-222	β-sheet
	226-229	β-sheet
	232-243	α-helix
α2	9-13	β-sheet
	20-30	α-helix
	34-39	β-sheet
	44-49	β-sheet
	66-68	β-sheet
	72-76	β-sheet
	81-100	α-helix
	107-124	α-helix
	131-139	β-sheet
	145-149	β-sheet
	155-163	β-sheet
	167-178	α-helix
	184-198	α-helix
	208-214	β-sheet
	219-220	β-sheet
	223-231	α-helix
α3	10-12	β-sheet
	18-29	α-helix
	33-38	β-sheet
	43-48	β-sheet

	66-68	β-sheet
	72-76	β-sheet
	80-100	α-helix
	107-124	α-helix
	131-139	β-sheet
	146-150	β-sheet
	157-164	β-sheet
	168-178	α-helix
	186-200	α-helix
	211-217	β-sheet
	224-227	β-sheet
	230-248	α-helix
α4	6-10	β-sheet
	17-27	α-helix
	31-36	β-sheet
	41-46	β-sheet
	62-65	β-sheet
	69-73	β-sheet
	78-97	α-helix
	104-121	α-helix
	128-136	β-sheet
	143-147	β-sheet
	154-161	β-sheet
	165-176	α-helix
	183-198	α-helix
	206-212	β-sheet
	217-219	β-sheet
	222-243	α-helix
α5	11-15	β-sheet
	22-32	α-helix
	36-41	β-sheet
	46-51	β-sheet
	66-70	β-sheet
	74-78	β-sheet
	83-102	α-helix
	109-120	α-helix
	138-146	β-sheet
	152-156	β-sheet
	162-170	β-sheet
	174-185	α-helix
	191-206	α-helix
	215-221	β-sheet
	226-228	β-sheet
	231-240	α-helix

α6	10-13	β-sheet
	19-30	α-helix
	35-39	β-sheet
	45-49	β-sheet
	62-66	β-sheet
	70-74	β-sheet
	79-98	α-helix
	105-122	α-helix
	129-137	β-sheet
	143-147	β-sheet
	154-161	β-sheet
	165-176	α-helix
	184-199	α-helix
	210-216	β-sheet
	221-223	β-sheet
	226-236	α-helix
α7	13-15	β-sheet
	22-32	α-helix
	36-41	β-sheet
	46-51	β-sheet
	67-70	β-sheet
	74-78	β-sheet
	83-102	α-helix
	109-124	α-helix
	133-141	β-sheet
	148-152	β-sheet
	160-166	β-sheet
	170-180	α-helix
	187-202	α-helix
	212-219	β-sheet
	224-227	β-sheet
	229-245	α-helix
β1	2-8	β-sheet
	12-16	β-sheet
	25-27	β-sheet
	34-37	β-sheet
	41-45	β-sheet
	52-69	α-helix
	76-90	α-helix
	95-103	β-sheet
	110-114	β-sheet
	120-122	β-sheet
	124-128	β-sheet
	132-143	α-helix

	149-166	α-helix
	174-180	β-sheet
	185-189	β-sheet
	191-200	α-helix
β2	2-8	β-sheet
	12-17	β-sheet
	25-27	β-sheet
	33-37	β-sheet
	41-45	β-sheet
	52-67	α-helix
	76-90	α-helix
	95-103	β-sheet
	109-113	β-sheet
	123-127	β-sheet
	131-142	α-helix
	148-165	α-helix
	173-179	β-sheet
	184-186	β-sheet
	194-201	α-helix
	210-213	β-sheet
	215-225	β-sheet
β3	9-14	β-sheet
	18-24	β-sheet
	32-34	β-sheet
	41-44	β-sheet
	48-52	β-sheet
	56-76	α-helix
	83-97	α-helix
	103-111	β-sheet
	119-123	β-sheet
	134-138	β-sheet
	142-152	α-helix
	159-175	α-helix
	184-190	β-sheet
	194-199	β-sheet
β4	3-8	β-sheet
	12-17	β-sheet
	26-27	β-sheet
	32-35	α-helix
	36-38	β-sheet
	42-46	β-sheet
	50-70	α-helix
	77-93	α-helix
	99-107	β-sheet

	114-118	β-sheet
	129-132	β-sheet
	136-147	α-helix
	153-170	α-helix
	178-184	β-sheet
	189-190	β-sheet
β5	2-8	β-sheet
	12-16	β-sheet
	25-26	β-sheet
	31-34	α-helix
	35-37	β-sheet
	41-45	β-sheet
	49-69	α-helix
	76-91	α-helix
	96-104	β-sheet
	110-114	β-sheet
	120-122	β-sheet
	124-128	β-sheet
	132-143	α-helix
	149-166	α-helix
	174-180	β-sheet
	185-186	β-sheet
	189-200	α-helix
β6	5-6	β-sheet
	11-16	β-sheet
	20-26	β-sheet
	34-36	β-sheet
	42-46	β-sheet
	50-54	β-sheet
	58-77	α-helix
	85-99	α-helix
	105-113	β-sheet
	120-124	β-sheet
	134-138	β-sheet
	142-152	α-helix
	168-184	α-helix
	193-199	β-sheet
	203-210	β-sheet
β7	10-16	β-sheet
	20-25	β-sheet
	33-35	β-sheet
	39-41	α-helix
	42-45	β-sheet
	49-53	β-sheet

	57-77	α-helix
	85-100	α-helix
	107-115	β-sheet
	121-125	β-sheet
	136-139	β-sheet
	143-154	α-helix
	162-179	α-helix
	187-193	β-sheet
	198-199	β-sheet
	207-216	α-helix

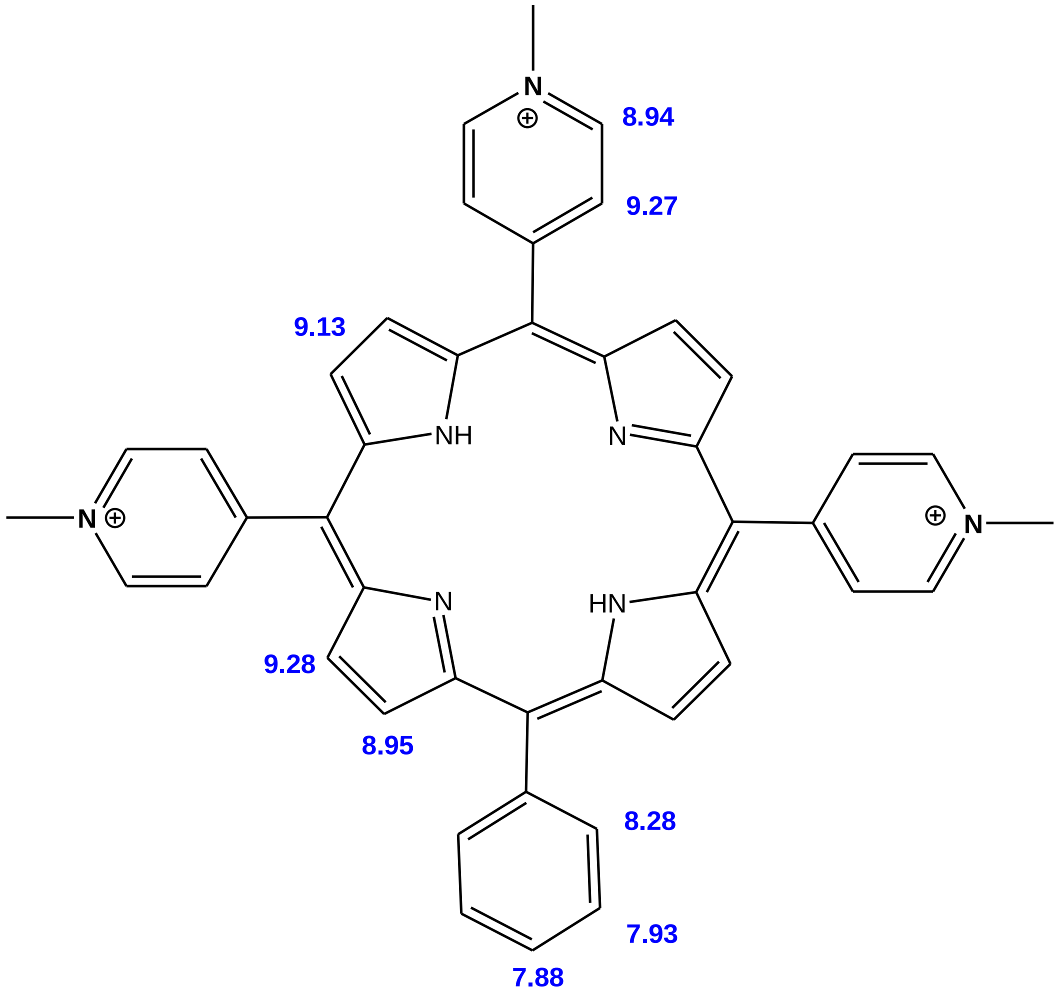

Figure S1. Structure of the **Tris-T4** reporting ^{1}H chemical shift assignments.

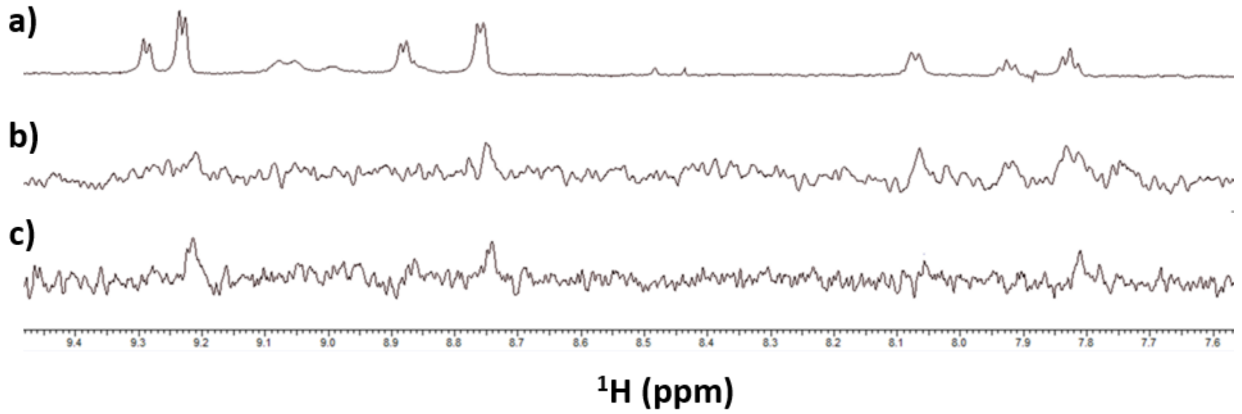

Figure S2. a) 1H NMR spectra of **Tris-T4** (100 μM) in the presence of $\alpha 3\Delta N$ y20S proteasome (500 nM); b) STD and c) WaterLOGSY spectra.

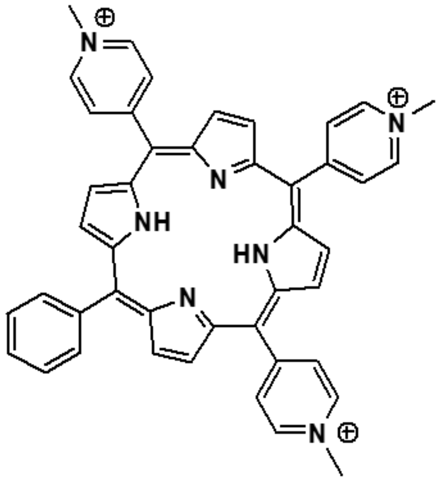

Figure S3. Structure of the calculated prevalent (79%;) ionic form of **Tris-T4** at cytoplasmic pH (7.2).

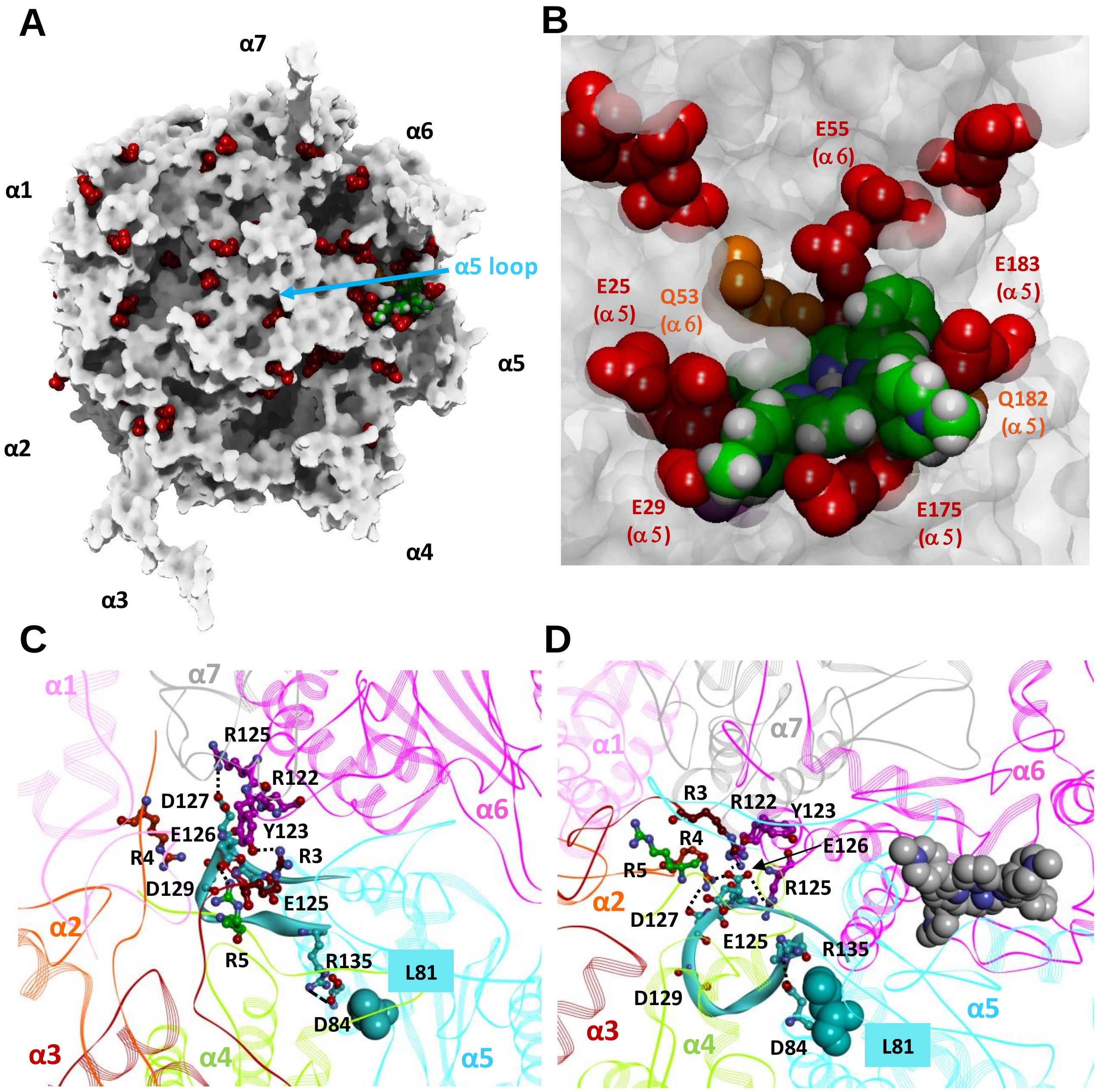

Figure S4. A-B: Selected docked complex of one **Tris-T4** molecule bound to human 20S (closed conformation). A) Top view: the protein is displayed as Connolly surface and colored in grey except for the negatively charged residues involved in ionic interactions with RPs which are displayed as CPK and colored in red. **Tris-T4** is colored by atom type (C: green; N: blue) and displayed as CPK. B) Close up view of the ligand binding site at the α5-α6 groove: interacting residues (non-bonded interaction energy lower than -2 kcal/mol) are evidenced as CPK and colored: negative = red; polar = orange. C-D: Close up view of the α5-loop interactions pattern of the closed proteasome 20S in the starting conformation (C) and upon one **Tris-T4** molecule binding at the α5-α6 groove (D). The α-ring is rendered in cartoons and labelled; amino acids involved in ionic interactions are displayed in ball and sticks and labelled. α5-L81 at the top of the allosteric pathway between distinct conformational states described by Ruschak et al.[13] is evidenced in cyan CPK. **Tris-T4** is shown in grey CPK.

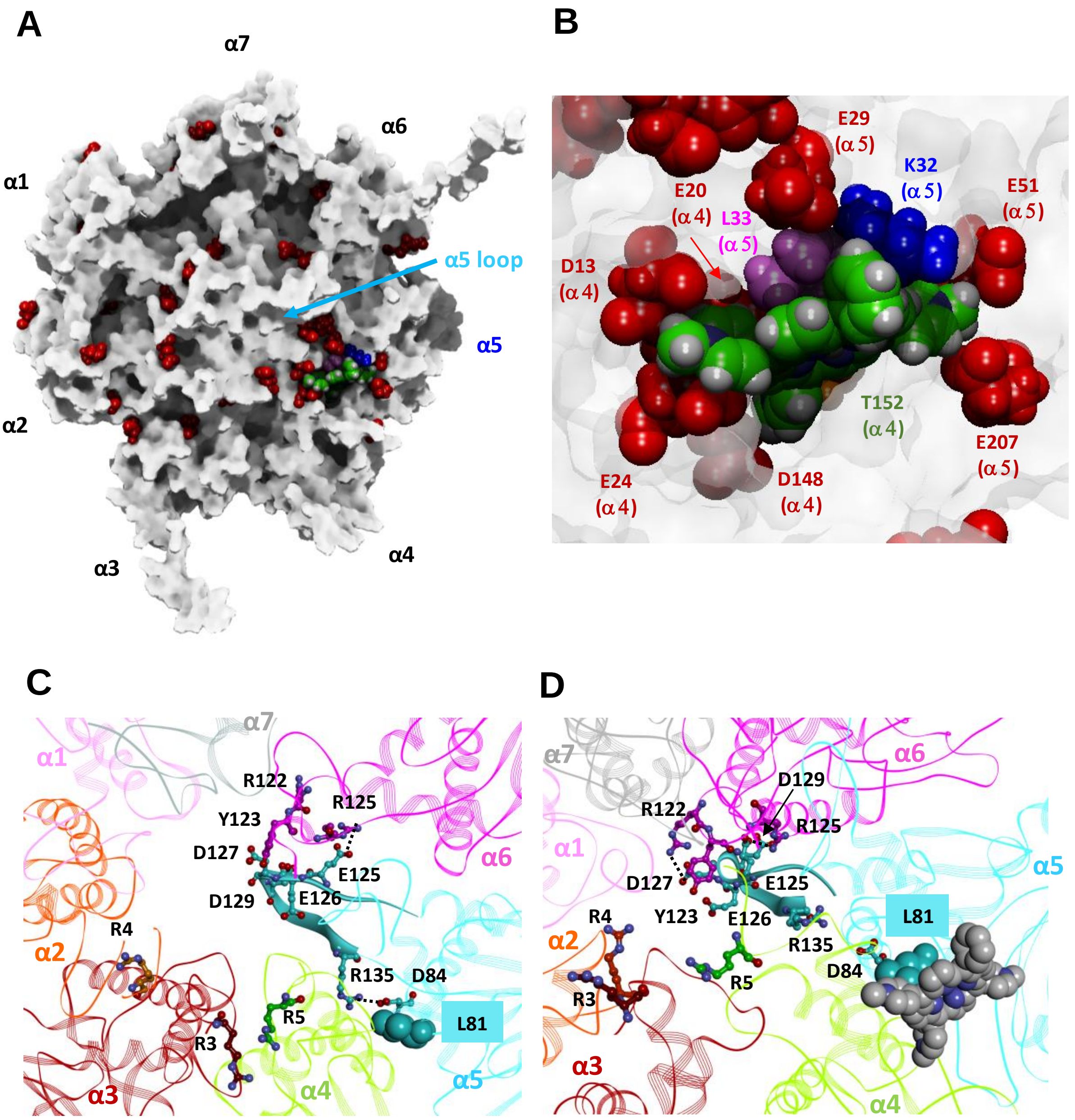

Figure S5. Selected docked complex of one **Tris-T4** molecule bound to human 20S (open conformation). A) Top view: the protein is displayed as Connolly surface and colored in grey except for the negatively charged residues involved in ionic interactions with RPs which are displayed as CPK and colored in red. **Tris-T4** is colored by atom type (C: green; N: blue) and displayed as CPK. B) Close up view of the ligand binding site at the α4-α5 groove: interacting residues (non-bonded interaction energy lower than -2 kcal/mol) are evidenced as CPK and colored: negative = red; positive = blue; polar = orange; hydrophobic = magenta. C-D: Close up view of the α5-loop interactions pattern of the open proteasome 20S in the starting conformation (C) and upon one **Tris-T4** molecule binding at the α4-α5 groove (D). α-ring is rendered in cartoons and labelled; amino acids involved in ionic interactions are displayed in ball and sticks and labelled. α5-L81 at the top of the allosteric pathway between distinct conformational states described by Ruschak et al.[13] is evidenced in cyan CPK. **Tris-T4** is shown in grey CPK.

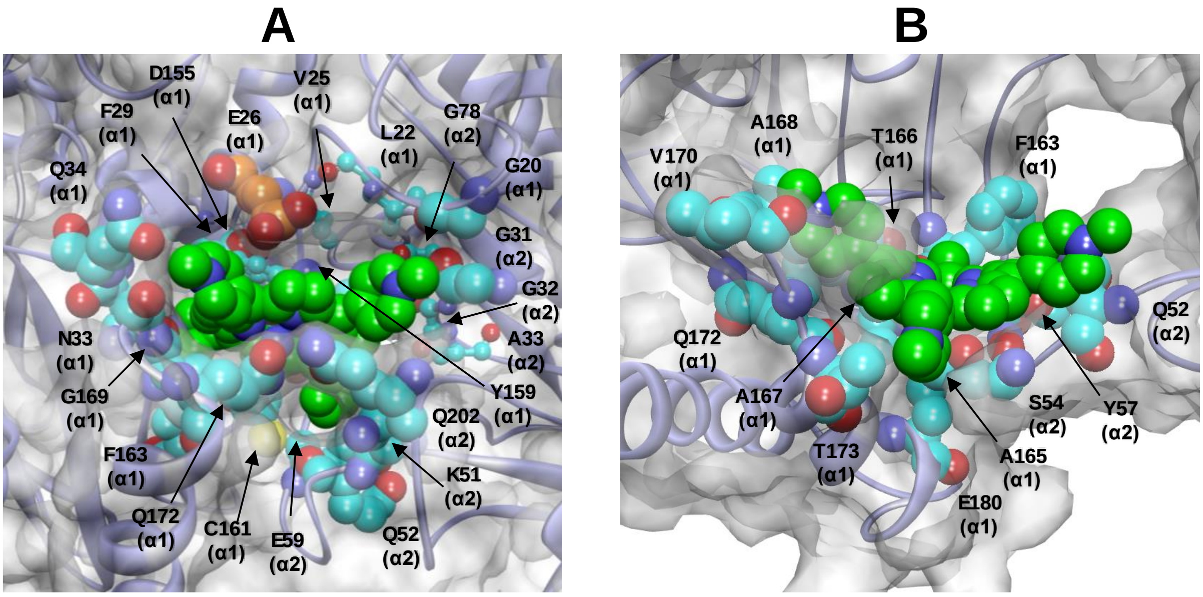

Figure S6. A-B: Close up view of the ligand binding site at the $\alpha 1$-$\alpha 2$ (A; closed) and $\alpha 1$-$\alpha 2$ (B; open) grooves. **Tris-T4** is colored by atom type (C: green; N: blue) and displayed as CPK. The protein is displayed as Connolly surface and colored in gray. The residues involved in interactions with **Tris-T4** are displayed in CPK and colored: orange (negative residues involved in ionic interactions with RPs) and cyan (residues not engaged in RP interactions). The residues involved in interactions with RPs hydrophobic motif are evidenced in ball&stick.

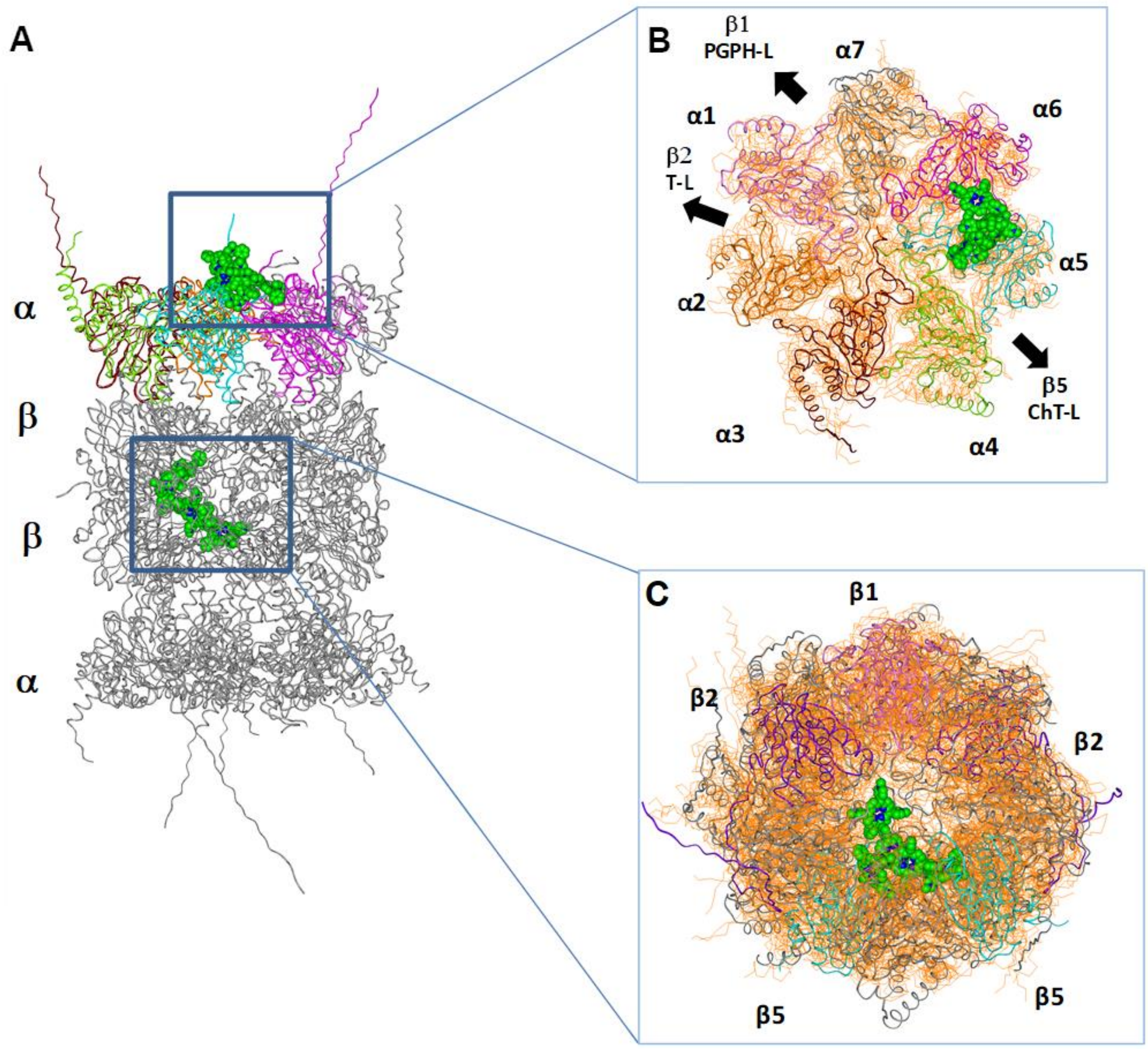

Figure S7. A) Dynamic docking results obtained for **Tris-T4** using as starting structure the $\alpha5$-$\alpha6$ groove of the closed conformation of human 20S proteasome. B) Top view of dynamic docking results at the first α-ring: the first five generated complexes (Table S4) show **Tris-T4** bound at the $\alpha5$-$\alpha6$ groove (although presenting a different binding mode with respect to the starting position). C) Top view of dynamic docking results without the first ring of α subunits: the last six generated complexes presented the ligand positioned either at the level of the first β-ring or at the interface between the first and the second β-ring (Table S4).The backbone of the starting complex is displayed as solid ribbons and colored in pink ($\alpha1$), orange ($\alpha2$), brown ($\alpha3$), light green ($\alpha4$), cyan ($\alpha5$), magenta ($\alpha6$), and gray ($\alpha7$, α subunits of the second ring and all β subunits). The backbone of the calculated complexes is displayed as line ribbons and colored in orange (B and C). The porphyrin ligands are colored by atom type (C: green and N: blue) and displayed as CPK. In B the α subunits and the catalytic β subunits are labeled. In C the catalytic β subunits are labeled and colored in pink (β1), violet (β2), and cyan (β5).

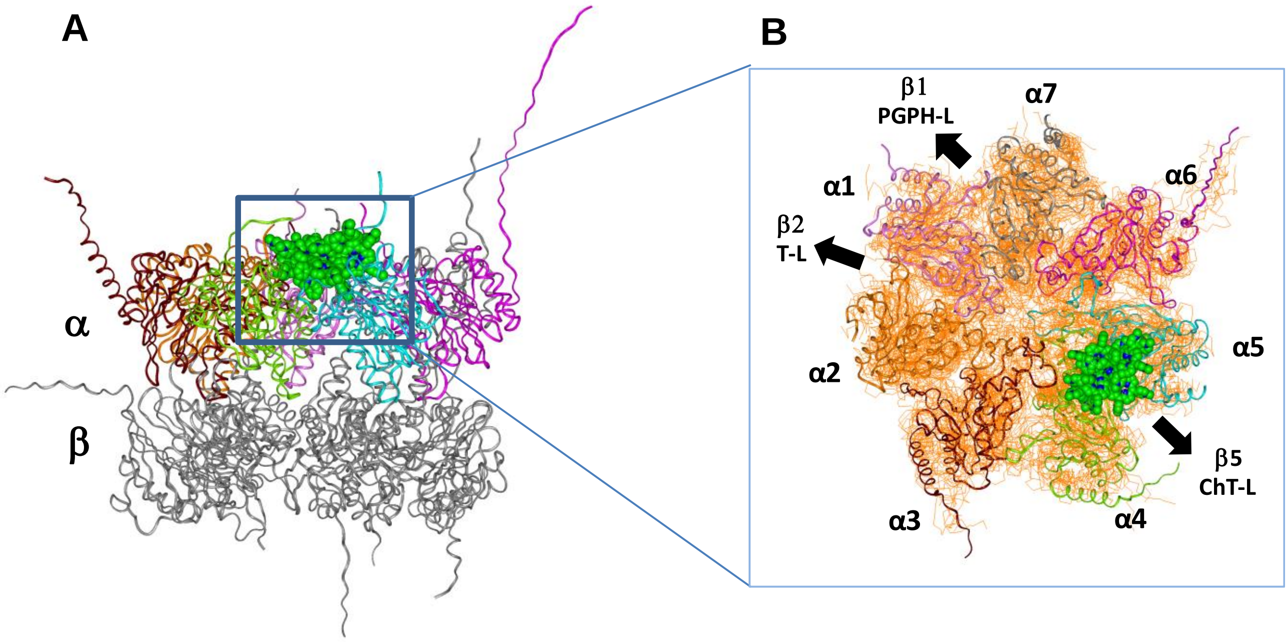

Figure S8. A) Dynamic docking results obtained for **Tris-T4** using as starting structure the α4-α5 groove of the open conformation of human 20S proteasome. B) Top view of dynamic docking results at the α-ring: all obtained complexes showed **Tris-T4** bound at the α4-α5 groove (Table S5), although assuming different binding poses with respect to the starting position). The backbone of the starting complex is displayed as solid ribbons and colored in pink (α1), orange (α2), brown (α3), light green (α4), cyan (α5), magenta (α6), and grey (α7, all β subunits). The backbone of the calculated complexes is displayed as line ribbons and colored in orange (B). The porphyrin ligands are colored by atom type (C: green and N: blue) and displayed as CPK. In B the α subunits and the catalytic β subunits are labeled.

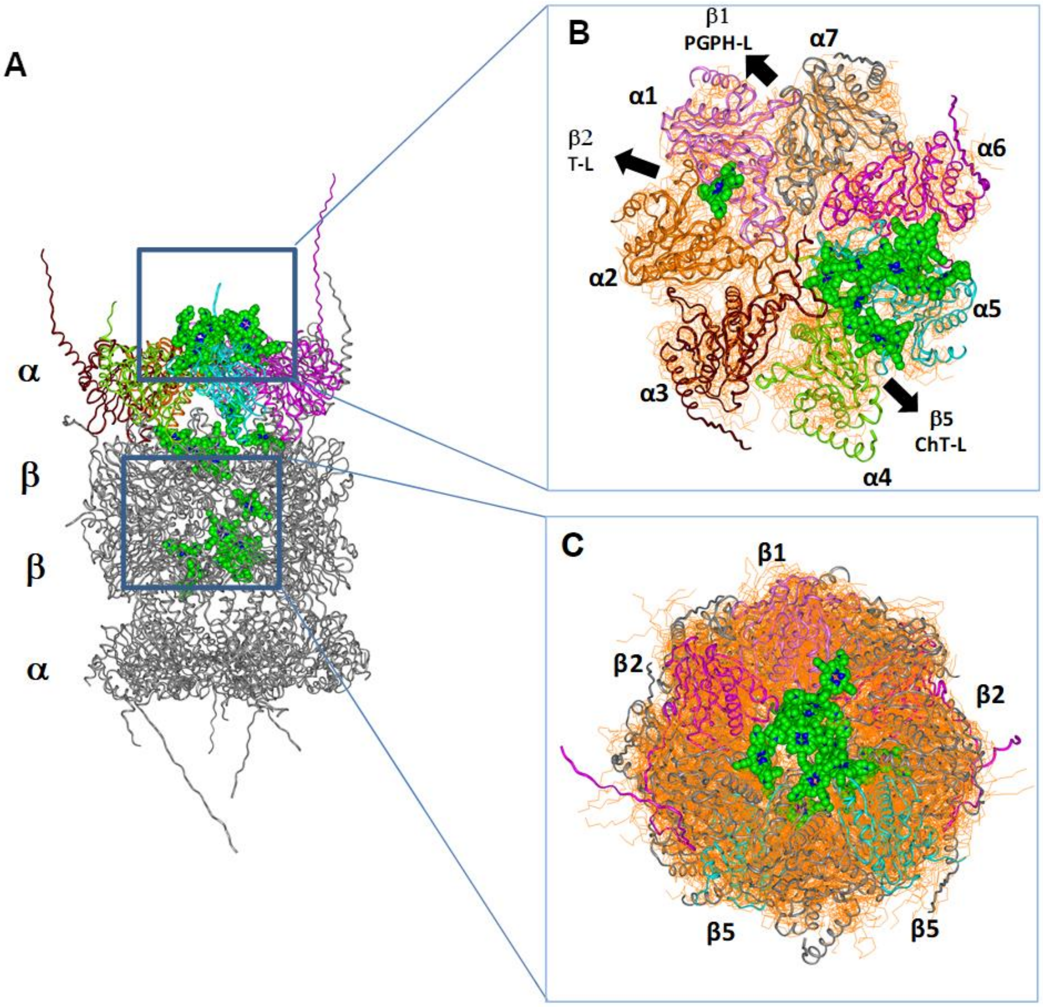

Figure S9. A) Dynamic docking results obtained for **Tris_T4** using as starting points the α1-α2. α4-α5. and α5-α6 grooves of the closed conformation of human 20S proteasome. B) Top view of dynamic docking results without the two rings of β subunits and the second ring of α subunits. C) Top view of dynamic docking results without the first ring of α subunits. The analysis of the whole of the generated complexes using the 20S closed conformation as starting structure showed that in four of them (2-5 in Table S6) one molecule of **Tris-T4** is placed at the level of the first β-ring (A and C). The other two molecules are localized on the α-ring, with one of the two always positioned at α5-α6 groove (B). Finally, in the subsequent four complexes (6-9 in Table S6), all three molecules move down, placing at the interface of the β rings or between the ⊚5 and ⊚6 subunits.

The backbone of the starting complex is displayed as solid ribbons and colored in pink (α1), orange (α2), brown (α3), light green (α4), cyan (α5), magenta (α6), and grey (α7, α subunits of the second ring and all β subunits). The backbone of the calculated complexes is displayed as line ribbons and colored in orange (B and C). The porphyrin ligands are colored by atom type (C: green and N: blue) and displayed as CPK. In B the α subunits and the catalytic β subunits are labeled. In C the catalytic β subunits are labeled and colored in pink (β1), violet (β2), and cyan (β5).

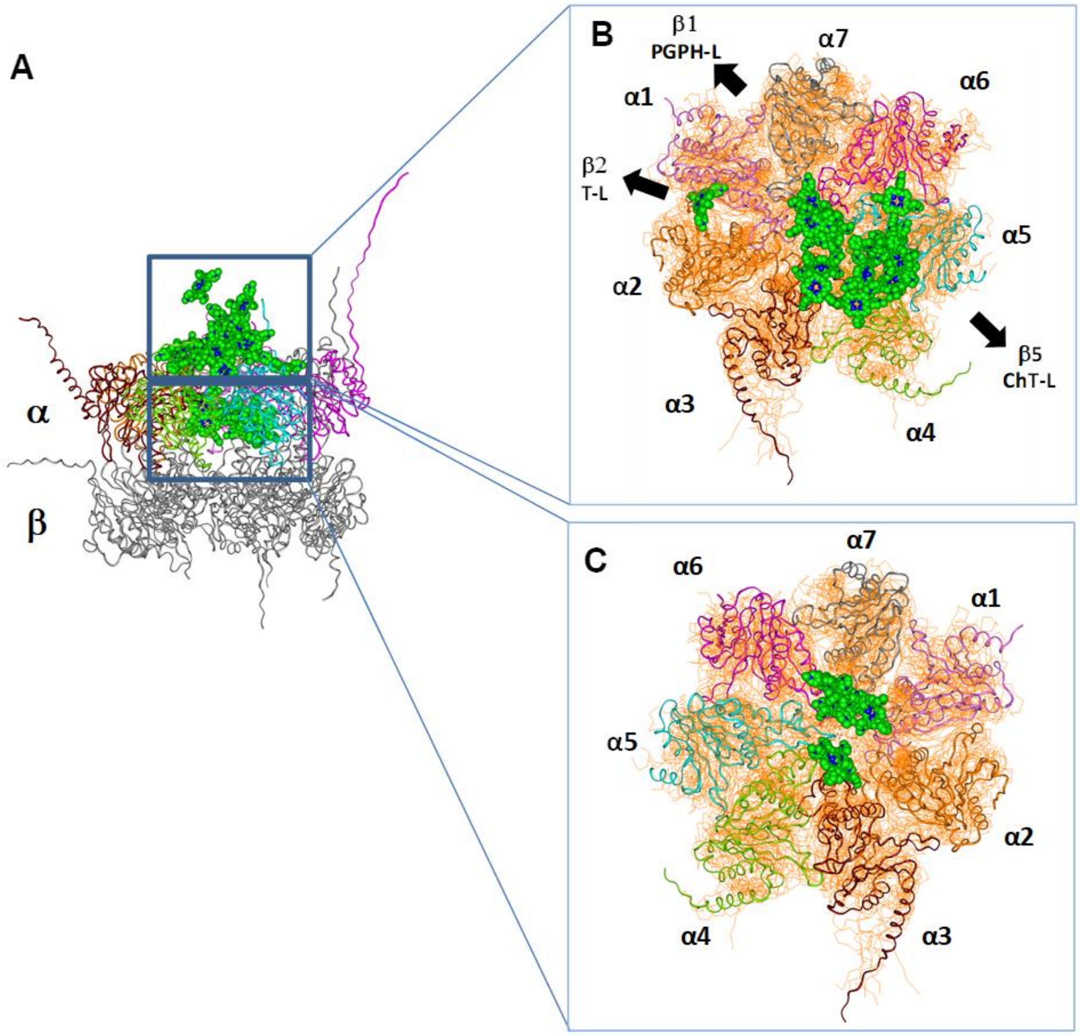

Figure S10. A) Dynamic docking results obtained for **Tris_T4** using as starting points the α1-α2. α4-α5. and α5-α6 grooves of the open conformation of human 20S proteasome. B) Top view of dynamic docking results without the ring of β subunits. C) Bottom view of dynamic docking results without the ring of β subunits. The ligand molecule bound to the ◎1-◎2 groove moves down the interior of the α-ring or toward the center of the protein (A and C). On the other hand, the other two molecules of **Tris-T4** in most solutions bind at ◎4-◎5 and ◎5-◎6 grooves although in some binding poses occupying the substrate channel between the ◎◎ and the ◎-ring or moving along the grooves toward the substrate gate. Interestingly, in four solutions one **Tris-T4** binds the negatively charged ◎5-loop (B).
The backbone of the starting complex is displayed as solid ribbons and colored in pink (α1), orange (α2), brown (α3), light green (α4), cyan (α5), magenta (α6), and grey (α7, α subunits of the second ring and all β subunits). The backbone of the calculated complexes is displayed as line ribbons and colored in orange (B and C). The porphyrin ligands are colored by atom type (C: green and N: blue) and displayed as CPK. In B the α subunits and the catalytic β subunits are labeled.

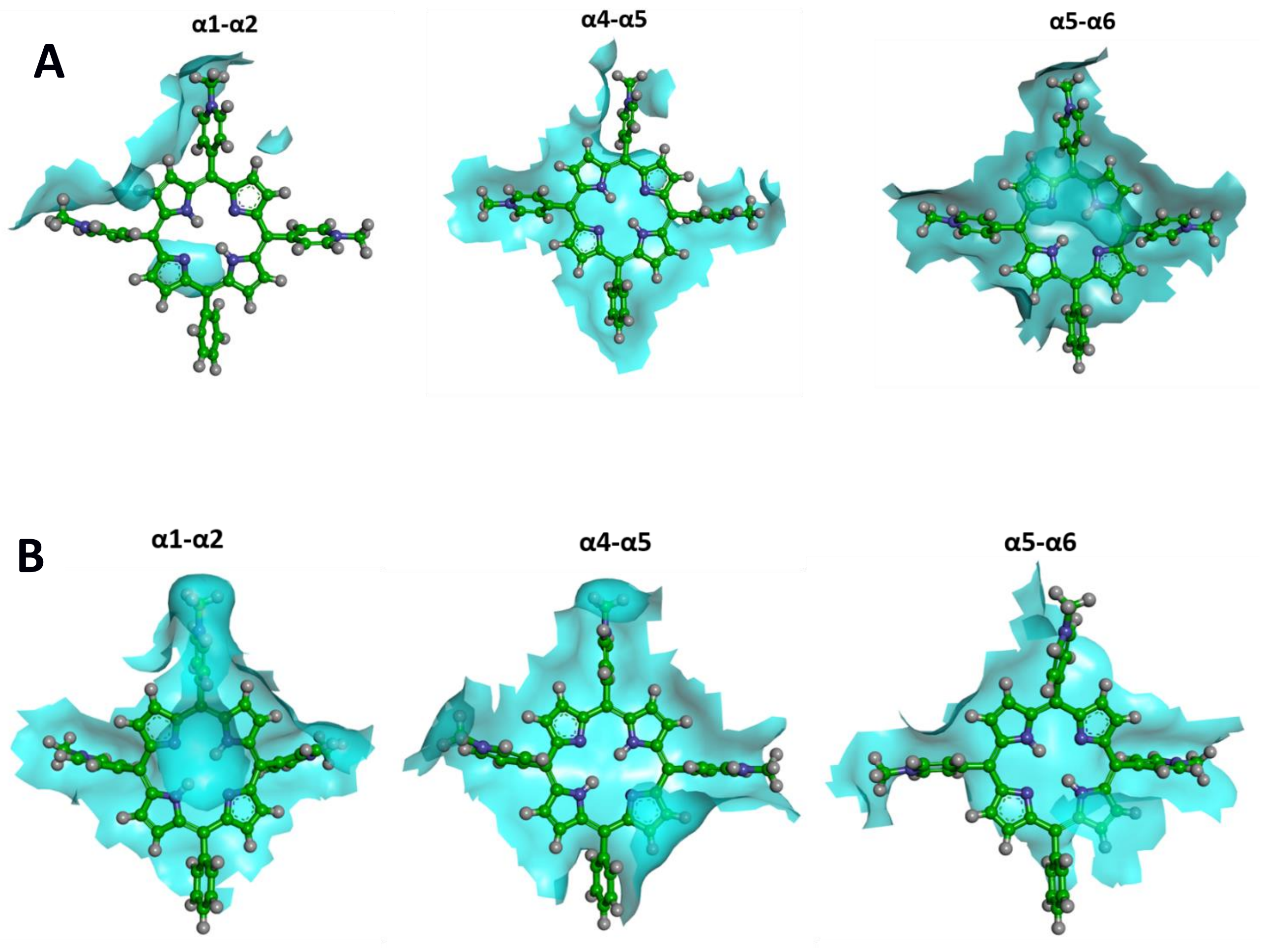

Figure S11. Solvent accessible surface (SASA) of the three **Tris-T4** molecules docked to the closed (A) and open (B) h20S conformation (view: from the interior to the exterior of the protein). SASA is coloured in cyan and displayed as solid surface; ligand molecules are coloured by atom types (C = green; N = blue; H= white).

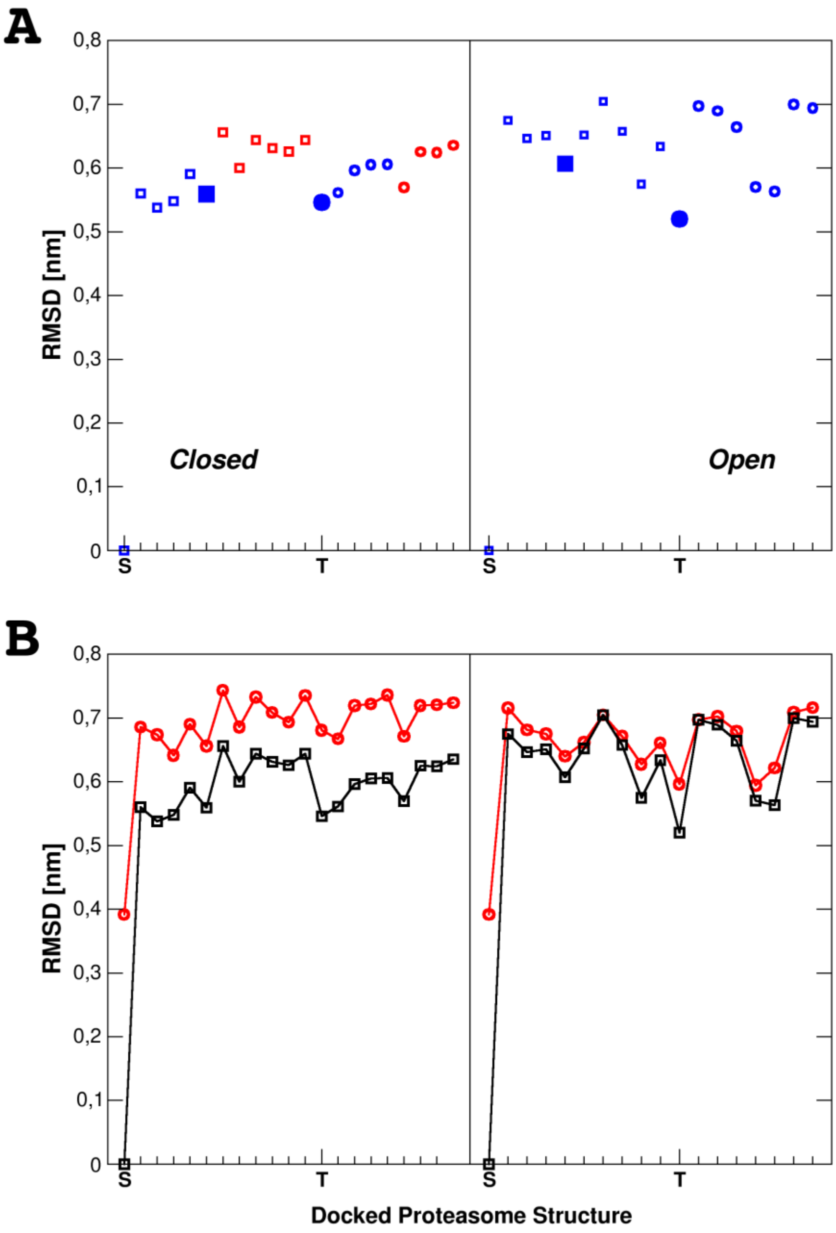

Figure S12. A) RMSD of the **Tris-T4**/20S complexes generated upon MC/SA docking calculations carried out on the closed (left) and open (right) states with respect to the starting protein conformation. Squared spots indicate single molecule docking (S on x-axis), circled spots indicate triple molecule docking (T on x-axis). Blue colour indicates complexes where at least one ligand molecule occupies superficial binding poses (α surface) whereas red colour is used for complexes where all ligand molecules occupy internal sites (α/β interface. β subunits). Full coloured spots specify selected binding poses. B) RMSD evolution is calculated with respect to the opposite 20S conformation (closed vs. open and open vs. closed; red line) and compared to those obtained using as reference structure the starting conformation (black line), as an index of divergence/similarity from the open/closed starting states. RMSDs are calculated on Cα atoms of the full-length (upper α and β rings) proteasome.

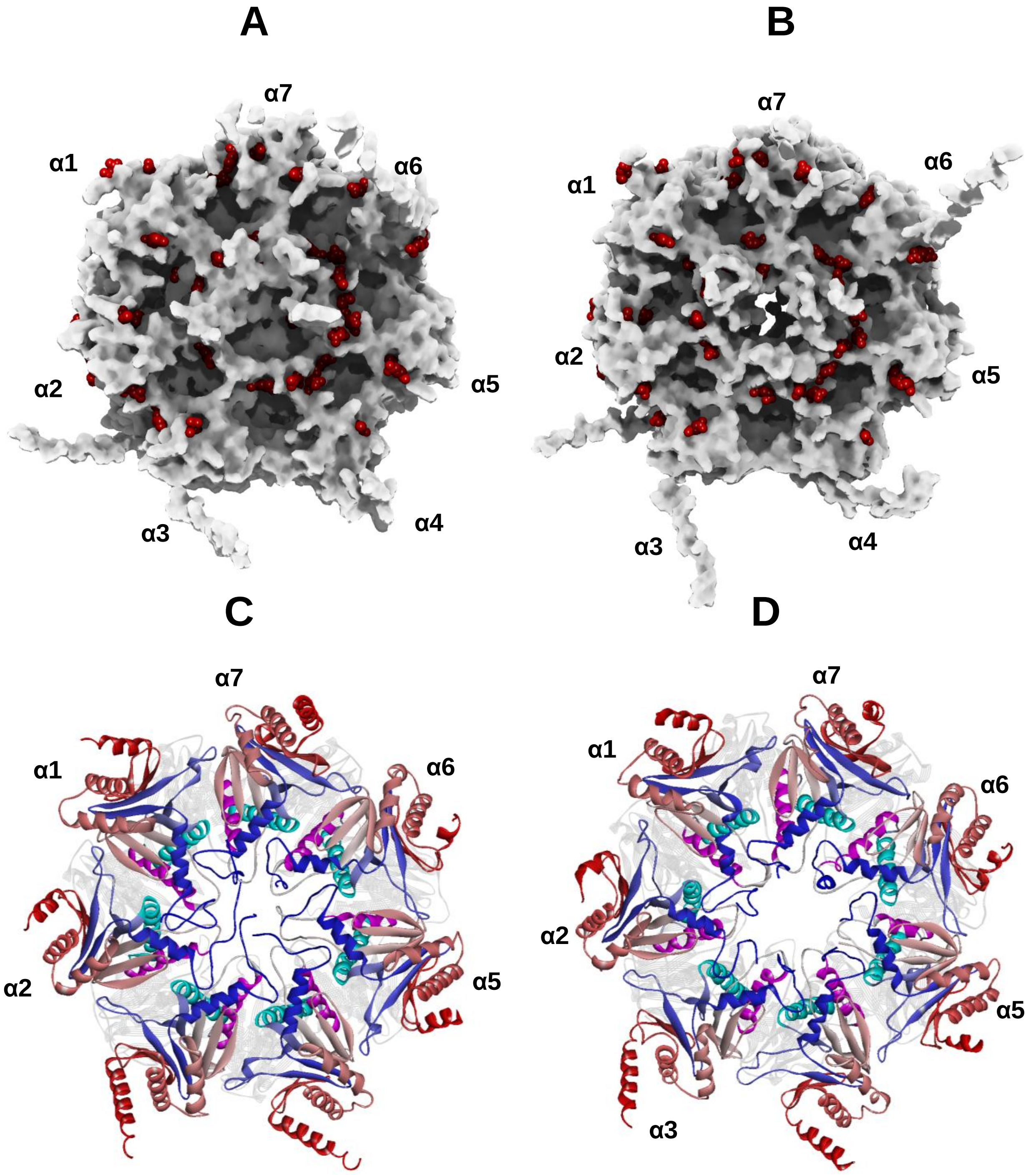

Figure S13. Top view of the human 20S proteasome structure in the closed (A, C; PDB ID: 4R3O) and open (B, D; PDB ID: 5T0J) conformation. A and B: the protein is displayed as Connolly surface and colored in gray; the negatively charged residues involved in ionic interactions with RPs (i.e., 19S, PA28α and PA200) are displayed as CPK and colored in red. C and D: the protein is displayed in ribbons and secondary structure elements of each subunit are sequentially colored from N- to C-terminal end (αH0= blue; αH1= cyan; αH2= magenta, αH3-αH5= light red to dark red).

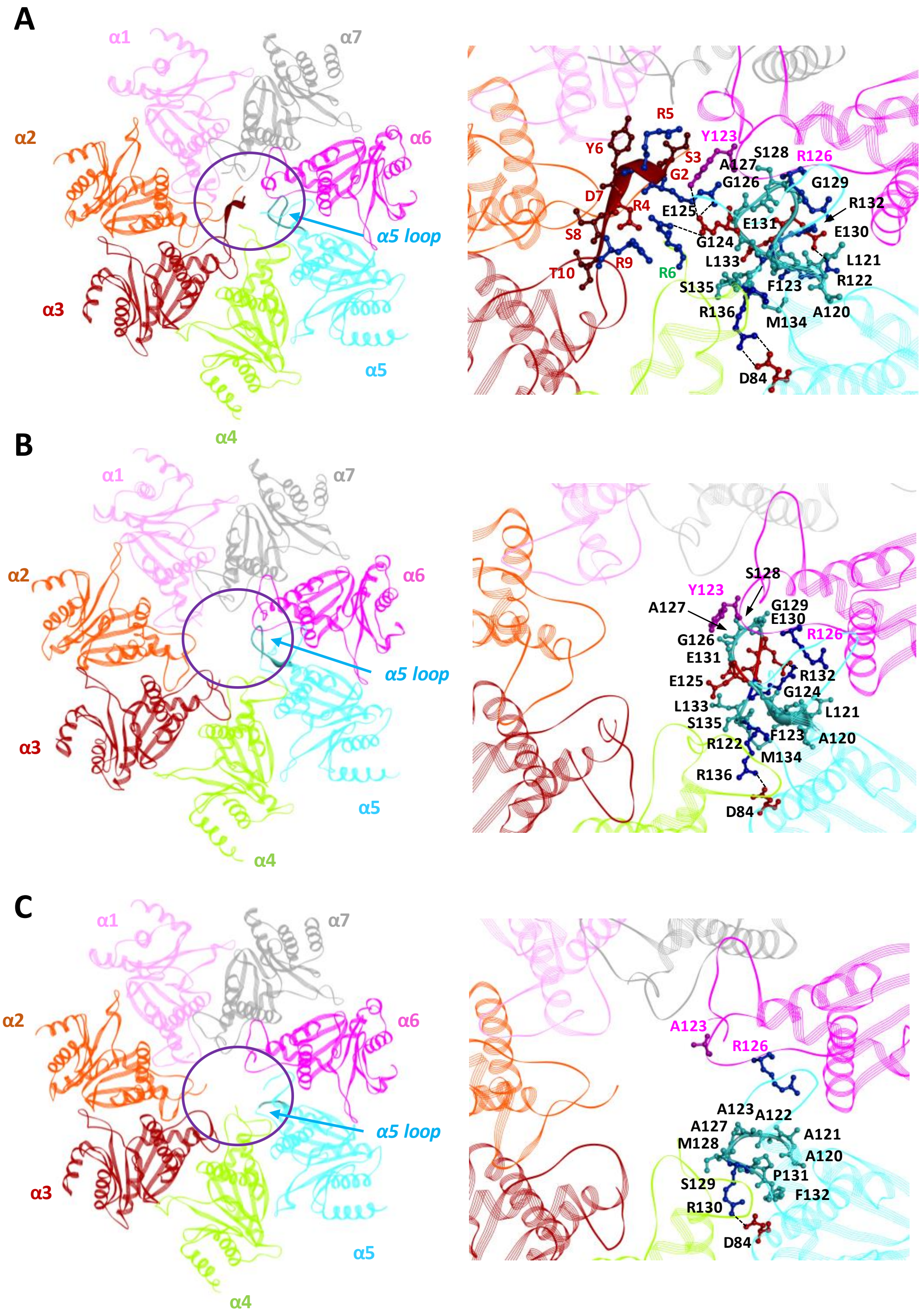

Figure S14. wt-yeast and $\alpha3\Delta N$-yeast 20S proteasome. Top-view of the α-ring (left) and close-up view of the gate (right). A) wt yeast 20S proteasome structures in closed state (access code: 1RYP); B) wt yeast 20S proteasome structures in open state (access code: 1Z7Q) and in C) mutant form $\alpha3\Delta N$ (access code: 1G0U) are shown in line cartoons. Purple ellipses define the proteasome gate and the $\alpha5$ loop is indicated and labelled. Amino acids of the N-terminal tails involved in the major interactions with the $\alpha5$ loop are displayed in ball and sticks and labelled, negatively and positively charged residues are coloured red and blue, respectively.

A

α5-Human	^{120}ALQFGEED-ADPGAMSR^{135}
α5-Yeast	^{120}ALRFGEGASGEERLMSR^{136}
α5-α3ΔN Yeast	^{120}AAAA---------AMSR^{136}

B

α6-Human	^{115}KTQIPTQRYGRRPYGVGLLI^{134}
α6-Yeast	KAQKNTQSYGGRPYGVGLLI
α6-α3ΔN Yeast	KAQKNTQSAGGRPYGVGLLI

Figure S15. Multiple sequence alignment of *α*5-loop (A) and *α*6-loop (B) of human, *yeast* and *α*3ΔN *yeast* 20S. Amino acids establishing salt bridges and charge-assisted hydrogen bonds are evidenced in cyan. Amino acids not conserved in the *α*3ΔN mutant are evidenced with black boxes.

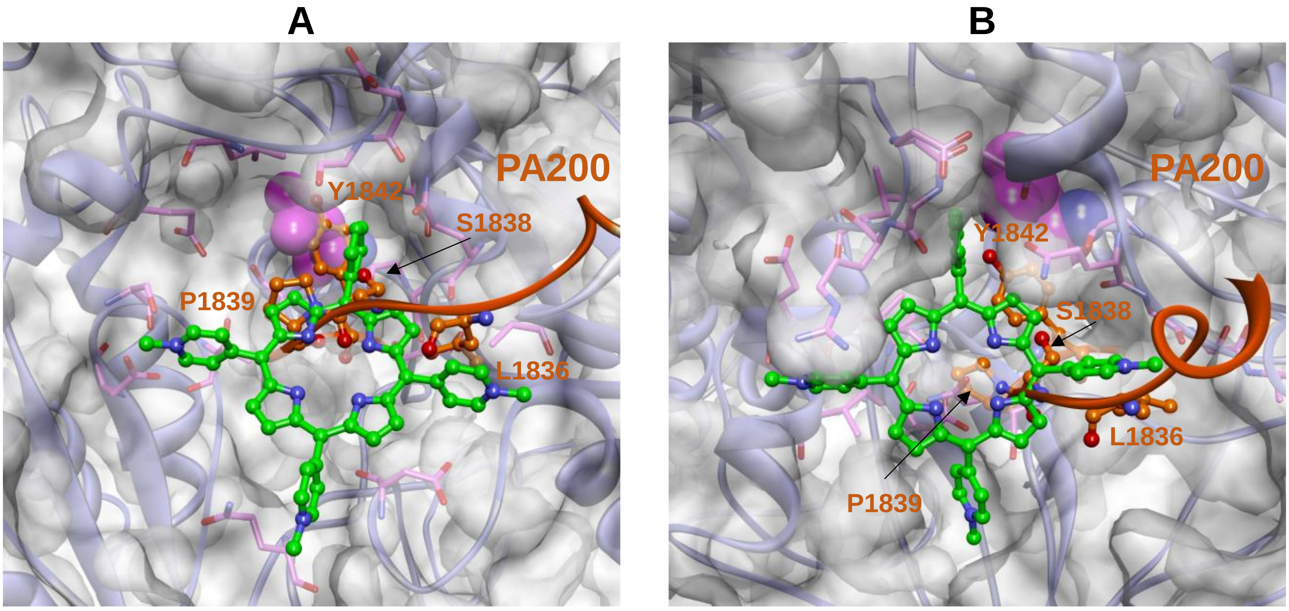

Figure S16. Structural overlap of the PA200 C-terminal tail with **Tris-T4** obtained by fitting the Cα atoms of the 20S α-ring of the **Tris-T4**/h20S best-docked complexes (three ligand molecules; h20S starting conformation: closed (A) and open (B)) on the Cα atoms of the 20S α-ring of the h20S structure in complex with PA200 (PDB ID: 6KWY). The PA200 C-terminal residues overlapping with **Tris-T4** are displayed in ball&stick and labelled (residues having at least one atom within a 2 Å radius from any given **Tris-T4** atom). For sake of clarity, only the h20S structure of the docked complex is displayed. Residues interacting with both **Tris-T4** and the PA200 residues are displayed in stick and coloured in pink. The vdW volume of L77 (α6) is shown in magenta.

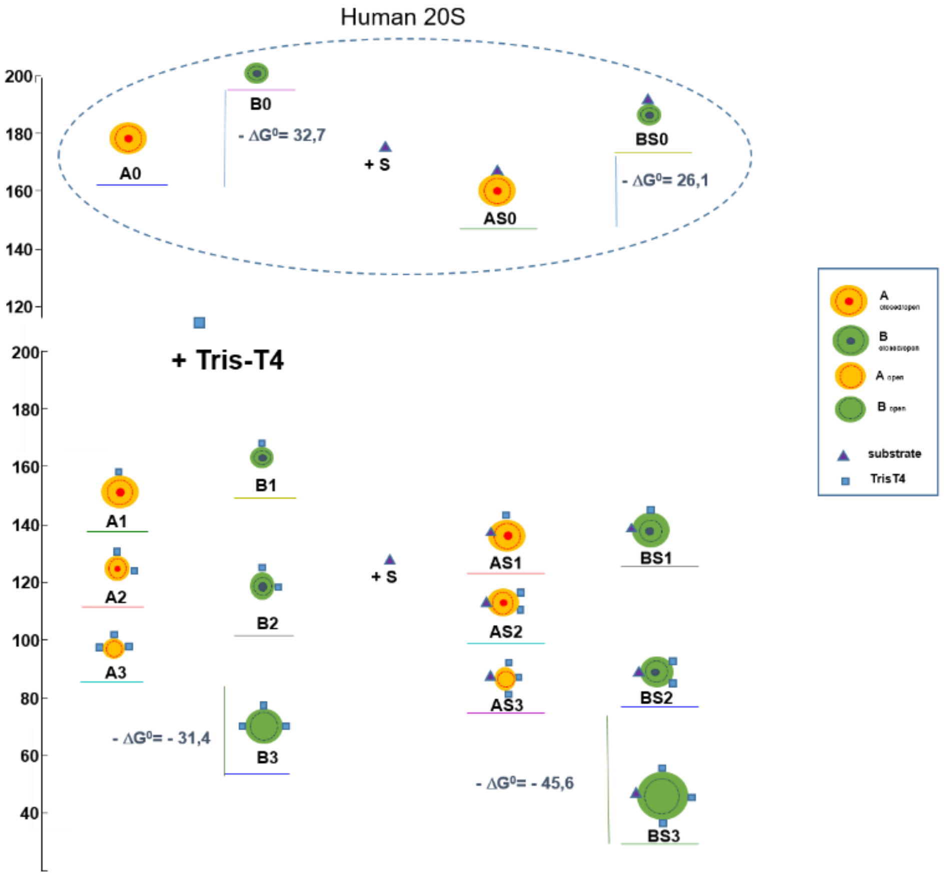

Figure S17. Graphical representation of standard Gibbs free energy difference (-ΔG0, as defined in the Experimental section) among the various **Tris-T4**- and substrate-bound states of h20S. The y-axis values (kJ/mol) are arbitrarily chosen. Circles represents different conformational states and the dimension is indicative of their abundance.

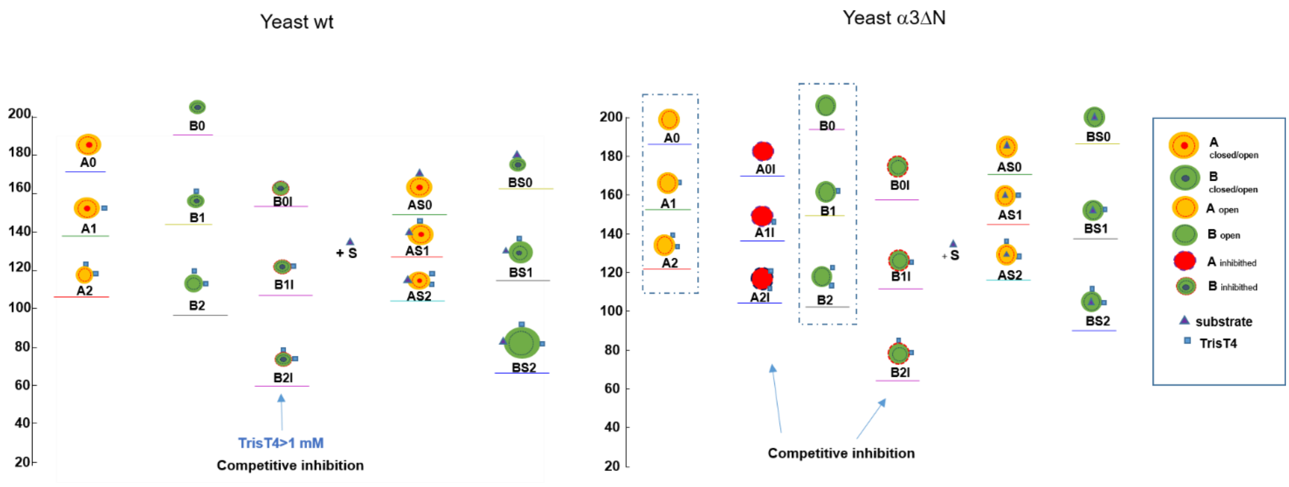

Figure S18. Standard Gibbs free energy difference (-ΔG_0, as defined in the Experimental section) among the various **Tris-T4** and substrate-bound states of y20S wt and y20S $\alpha3\Delta N$ mutant. The y-axis values are in kJ/mol arbitrarily chosen, but the energy differences correspond to those resulting from parameters reported in Table 1. Circles represents different conformational states and the dimension is indicative of their abundance.

References

(1) Dewar, M. J. S.; Thiel, W. Ground states of molecules. 38. The MNDO method. Approximations and parameters. *J. Am. Chem. Soc.* **1977**, *99* (15), 4899–4907. https://doi.org/10.1021/ja00457a004.

(2) Fletcher, R. (Roger). *Practical methods of optimization*; Wiley, 1987.

(3) Stewart, J. J. P. Optimization of parameters for semiempirical methods VI: more modifications to the NDDO approximations and re-optimization of parameters. *J. Mol. Model.* **2013**, *19* (1), 1–32. https://doi.org/10.1007/s00894-012-1667-x.

(4) Baker, J. An algorithm for the location of transition states. *J. Comput. Chem.* **1986**, *7* (4), 385–395. https://doi.org/10.1002/jcc.540070402.

(5) Dato, A. Di; Cunsolo, A.; Persico, M.; Santoro, A. M.; D'Urso, A.; Milardi, D.; Purrello, R.; Stefanelli, M.; Paolesse, R.; Tundo, G. R.; Sbardella, D.; Fattorusso, C.; Coletta, M. Electrostatic Map Of Proteasome α-Rings Encodes The Design of Allosteric Porphyrin-Based Inhibitors Able To Affect 20S Conformation By Cooperative Binding. *Sci. Rep.* **2017**, *7* (1), 17098. https://doi.org/10.1038/s41598-017-17008-7.

(6) Chen, S.; Wu, J.; Lu, Y.; Ma, Y.-B.; Lee, B.-H.; Yu, Z.; Ouyang, Q.; Finley, D. J.; Kirschner, M. W.; Mao, Y. Structural basis for dynamic regulation of the human 26S proteasome. *Proc. Natl. Acad. Sci. U. S. A.* **2016**, *113* (46), 12991–12996. https://doi.org/10.1073/pnas.1614614113.

(7) Senderowitz, H.; Guarnieri, F.; Still, W. C. A Smart Monte Carlo Technique for Free Energy Simulations of Multiconformational Molecules. Direct Calculations of the Conformational Populations of Organic Molecules. *J. Am. Chem. Soc.* **1995**, *117* (31), 8211–8219. https://doi.org/10.1021/ja00136a020.

(8) Ding, H.; Karasawa, N.; Goddard, W. A. Atomic level simulations on a million particles: The cell multipole method for Coulomb and London nonbond interactions. *J. Chem. Phys.* **1992**, *97* (6), 4309–4315. https://doi.org/10.1063/1.463935.

(9) Steinbach, P. J.; Brooks, B. R. New spherical-cutoff methods for long-range forces in macromolecular simulation. *J. Comput. Chem.* **1994**, *15* (7), 667–683. https://doi.org/10.1002/jcc.540150702.

(10) Davis, I. W.; Leaver-Fay, A.; Chen, V. B.; Block, J. N.; Kapral, G. J.; Wang, X.; Murray, L. W.; Arendall, W. B.; Snoeyink, J.; Richardson, J. S.; Richardson, D. C. MolProbity: all-atom contacts and structure validation for proteins and nucleic acids. *Nucleic Acids Res.* **2007**, *35* (Web Server), W375–W383. https://doi.org/10.1093/nar/gkm216.

(11) Pei, J.; Kim, B.-H.; Grishin, N. V. PROMALS3D: a tool for multiple protein sequence and structure alignments. *Nucleic Acids Res.* **2008**, *36* (7), 2295–2300. https://doi.org/10.1093/nar/gkn072.

(12) Chovancova, E.; Pavelka, A.; Benes, P.; Strnad, O.; Brezovsky, J.; Kozlikova, B.; Gora, A.; Sustr, V.; Klvana, M.; Medek, P.; Biedermannova, L.; Sochor, J.; Damborsky, J.

CAVER 3.0: A Tool for the Analysis of Transport Pathways in Dynamic Protein Structures. *PLoS Comput. Biol.* **2012**, *8* (10), e1002708. https://doi.org/10.1371/journal.pcbi.1002708.

(13) Ruschak, A. M.; Kay, L. E. Proteasome allostery as a population shift between interchanging conformers. *Proc. Natl. Acad. Sci. U. S. A.* **2012**, *109* (50), E3454-62. https://doi.org/10.1073/pnas.1213640109.
